# Next generation sequencing of extraskeletal myxoid chondrosarcoma

**DOI:** 10.18632/oncotarget.15568

**Published:** 2017-02-21

**Authors:** Elizabeth J. Davis, Yi-Mi Wu, Dan Robinson, Scott M. Schuetze, Laurence H. Baker, Jyoti Athanikar, Xuhong Cao, Lakshmi P. Kunju, Arul M. Chinnaiyan, Rashmi Chugh

**Affiliations:** ^1^ Department of Internal Medicine, University of Michigan, Ann Arbor, MI, USA; ^2^ Michigan Center for Translational Pathology, University of Michigan, Ann Arbor, MI, USA

**Keywords:** extraskeletal myxoid chondrosarcoma, next generation sequencing, *EWSR1-NR4A3*

## Abstract

Extraskeletal myxoid chondrosarcoma (EMC) is an indolent translocation-associated soft tissue sarcoma with a high propensity for metastases. Using a clinical sequencing approach, we genomically profiled patients with metastatic EMC to elucidate the molecular biology and identify potentially actionable mutations. We also evaluated potential predictive factors of benefit to sunitinib, a multi-targeted tyrosine kinase inhibitor with reported activity in a subset of EMC patients. Between January 31, 2012 and April 15, 2016, six patients with EMC participated in the clinical sequencing research study. High quality DNA and RNA was isolated and matched normal samples underwent comprehensive next generation sequencing (whole or OncoSeq capture exome of tumor and normal, tumor PolyA+ and capture transcriptome). The expression levels of sunitinib targeted-kinases were measured by transcriptome sequencing for *KDR, PDGFRA/B, KIT, RET, FLT1*, and *FLT4*. The previously reported *EWSR1-NR4A3* translocation was identified in all patient tumors; however, other recurring genomic abnormalities were not detected. *RET* expression was significantly greater in patients with EMC relative to other types of sarcomas except for liposarcoma (p<0.0002). The folate receptor was overexpressed in two patients. Our study demonstrated that similar to other translocation-associated sarcomas, the mutational profile of metastatic EMC is limited beyond the pathognomonic translocation. The clinical significance of *RET* expression in EMC should be explored. Additional pre-clinical investigations of EMC may help elucidate molecular mechanisms contributing to EMC tumorigenesis that could be translated to the clinical setting.

## INTRODUCTION

Extraskeletal myxoid chondrosarcoma (EMC) is a translocation-associated soft tissue sarcoma (STS) accounting for less than 3% of all STS. In most EMCs, the characteristic translocation juxtaposes *NR4A3* on chromosome 9 with EWSR1 on chromosome 22, t(9;22). Other translocations, including *NR4A3* fused to TATA binding protein-associated factor 15 (*TAF15*), have been described [1]. Clinical presentation of EMC is generally an enlarging soft tissue mass in the proximal extremity of a man in his fourth to sixth decade of life [2]. EMCs demonstrate a strong tendency for local recurrence (37-48%) and metastatic disease (50%), usually pulmonary. Despite the high propensity for recurrence and/or metastases, the growth rate is fairly indolent with 5, 10, and 15-year survival rates in 82-90%, 65-70%, and 58-60% of cases, respectively [3, 4]. These rates represent much longer survival than for other STS supporting the unique biology of EMC. Investigating the molecular mechanisms of EMC is needed to better understand this unique sarcoma subtype and to develop a therapeutic approach for metastatic disease.

Effective treatment for EMC is wide local excision. Cytotoxic chemotherapy has no role in localized disease and has limited benefit in metastatic disease. Multiple case series of EMC patients treated with a breadth of therapies including doxorubicin, dacarbazine, cyclophosphamide, actinomycin-D, β-interferon, ifosfamide, gemcitabine and docetaxel showed no measurable responses [4, 5]. In contrast, a retrospective evaluation of 11 patients with progressive, metastatic EMC reported responses to anthracycline-based chemotherapy suggesting that there may be a subset of EMC patients who derive benefit from cytotoxic treatment [6].

Recently, there has been reported benefit in EMC patients treated with sunitinib in a small case series evaluating drug activity and correlating tumor response with molecular and biochemical characteristics of the tumors. Of 10 patients treated, 8 patients who showed clinical benefit (6 partial responses, 2 stable disease > 3-6+ months) harbored the *EWSR1-NR4A3* translocation [7]. Two patients who progressed had a *TAF-15-NR4A3* translocation, t(9;17). This variant translocation as well as a *NR4A3-TCF12/HTF4*, t(9;15) translocation were previously reported [8, 9]. Immunohistochemical and biochemical analyses did not reveal any significant predictive markers; however, analysis of receptor tyrosine kinase (RTK) activity demonstrated elevated expression and activation of RET, a known target of sunitinib [7].

There is a need to better understand EMC biology and identify therapeutic approaches. Here, we utilized a clinical sequencing approach to genomically profile patients with metastatic EMC to elucidate the biology of EMC and identify potentially actionable mutations or predictive factors of sunitinib response.

## RESULTS

**Figure 1:**
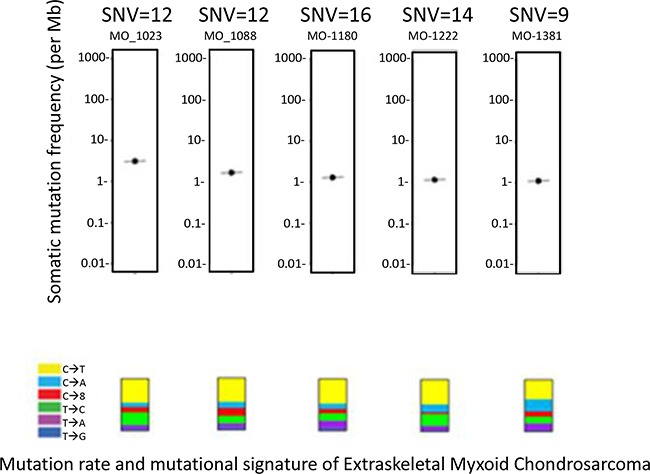
Mutation rate and mutational signature All mutations were of unknown significance and not known to be clinically actionable based on Precision Medicine Tumor Board review.

**Figure 2:**
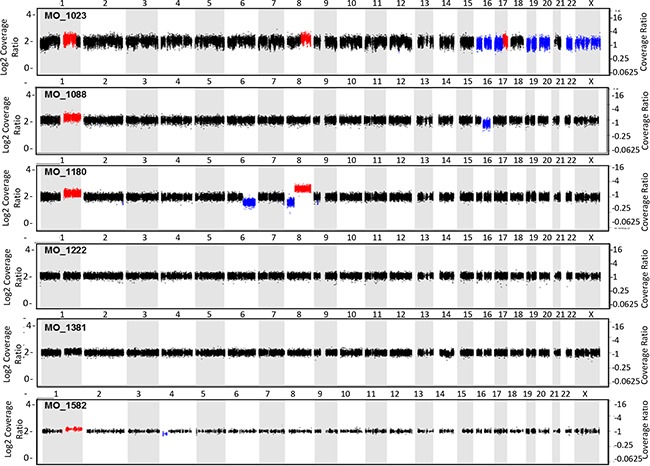
Integrative sequencing and mutational analysis of EMC Copy number alterations generated by whole exome sequencing of tumor and matched.

Six patients with EMC were enrolled on MI-ONCOSEQ. The median age of the patients at diagnosis was 44 years old (range 33-65) with median follow-up of 10 years (2-23 years). All patients are male and alive with disease. Detailed clinical information is presented in Table 1. Five out of six biopsy samples passed all quality control measures; one had limited, low quality RNA yield. The previously reported *EWSR1-NR4A3* translocation was identified in five of six patient tumors. Gene fusions could not be detected in the biopsy sample with poor RNA quality; however the *EWSR1-N4A3* translocation was detected in the patient's archived primary specimen using fluorescent in situ hybridization (FISH). Somatic point mutations were identified (total calls ranging from 2-26) but were of unknown significance (Figure 1). The number of somatic mutations was in the lower range of the number of mutations detected in other soft tissue sarcomas sequenced by the MI-ONCOSEQ program. Exome copy number analysis did not detect clinically significant focal amplifications or deletions; however, copy gains of chr1q and 8q, copy losses chr6q and 8p, and one copy loss of *ARID1B, MYB, CDKN2A, CDKN2B* among others were observed (Figure 2). No clinically significant indels or germline mutations were identified.

**Table 1:** Patient, tumor, and treatment characteristics

Patient	Age at diagnosis	Sex	Race	Site of primary disease	Disease stage at diagnosis	Biopsy site	Treatment history	Survival since diagnosis (years)
**MO-1023**	51	M	C	Thigh	Metastatic	Lung	Surgery, radiation	7+
**MO-1088**	65	M	C	Arm	Metastatic	Lung	Surgery	20+
**MO-1180**	56	M	C	Thigh	Metastatic	Gluteus	Radiation	4+
**MO-1222**	33	M	C	Thigh	Metastatic	Thigh	Surgery, Vincristine, doxorubicin, cyclophosphamide × 1 cycle, Ifosfamide, etoposide × 1 cycle, Dacarbazine × 4 cycles, R1507 × 8 months	13+
**MO-1381**	34	M	C	Thigh	Localized, 15 yrs until metastasis	Lung	Surgery, radiation, Cyclophosphamide × 24 months with Rapamycin × 55 months	23+
**MO-1582**	36	M	C	Lung	Metastatic	Lung	Pazopanib × 2 months, Doxorubicin × 3 months	2+

**Figure 3:**
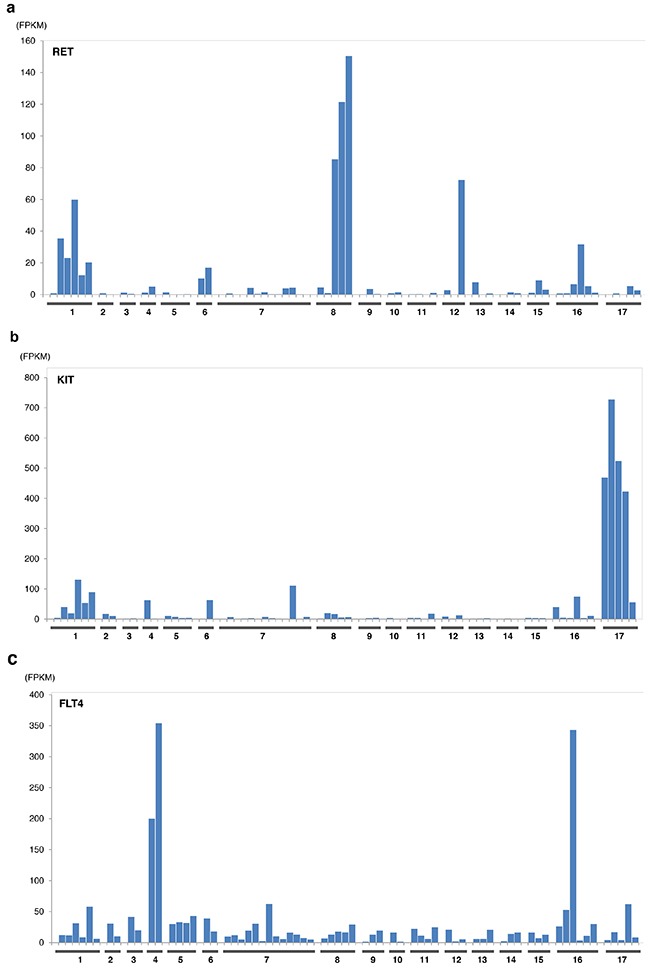
Expression of sunitinib target genes in EMC as compared to MIONCOSEQ sarcoma cohort Histologic subtypes evaluated: (1) Extraskeletal myxoid chondrosarcoma, (2) Chondrosarcoma, (3) Alveolar soft part sarcoma, (4) Angiosarcoma, (5) Desmoplastic round cell tumor, (6) Ewing's sarcoma, (7) Leiomyosarcoma, (8) Liposarcoma, (9) Myoepithelioma, (10) Osteosarcoma, (11) Pleomorphic sarcoma, (12) Rhabdomyosarcoma, (13) Sarcomatoid carcinoma, (14) Solitary fibrous tumor, (15) Synovial sarcoma, (16) Other sarcomas, (17) GIST. **a**. RET expression, **b**. KIT expression, **c**. Flt4 expression. Histologic subtypes evaluated: (1) Extraskeletal myxoid chondrosarcoma, (2) Chondrosarcoma, (3) Alveolar soft part sarcoma, (4) Angiosarcoma, (5) Desmoplastic round cell tumor, (6) Ewing's sarcoma, (7) Leiomyosarcoma, (8) Liposarcoma, (9) Myoepithelioma, (10) Osteosarcoma, (11) Pleomorphic sarcoma, (12) Rhabdomyosarcoma, (13) Sarcomatoid carcinoma, (14) Solitary fibrous tumor, (15) Synovial sarcoma, (16) Other sarcomas, (17) GIST. **d**. Flt1 expression, **e**. KDR expression, **f**. PDGFRα expression. Histologic subtypes evaluated: (1) Extraskeletal myxoid chondrosarcoma, (2) Chondrosarcoma, (3) Alveolar soft part sarcoma, (4)Angiosarcoma, (5) Desmoplastic round cell tumor, (6) Ewing's sarcoma, (7) Leiomyosarcoma, (8) Liposarcoma, (9) Myoepithelioma, (10) Osteosarcoma, (11) Pleomorphic sarcoma, (12) Rhabdomyosarcoma, (13) Sarcomatoid carcinoma, (14) Solitary fibrous tumor, (15) Synovial sarcoma, (16) Other sarcomas, (17) GIST. **g**. PDGFRβ expression.

The expression levels of sunitinib targeted-kinases were measured by transcriptome sequencing for *PDGFRA/B, KIT, RET, FLT1(VEGFR1), KDR (VEGFR2)*, and *FLT4(VEGFR3)* (Figure 3) for patient cohort with EMC and other sarcoma histologies. Only *RET* expression was significantly greater in patients with EMC relative to other types of sarcomas excluding liposarcoma (p<0.0002, by student t-test). Outlier overexpression of folate receptor was also observed in two EMC patients. Additional detailed molecular profiling information is available in Supplementary Materials.

## DISCUSSION

Given the advances and accessibility of next-generation sequencing technology, current practice of “precision cancer medicine” is increasing attempting to match genetic aberrations in individual patients with molecularly targeted therapies. It remains unclear if this approach will result in clinically meaningful treatment. Thus far, reports of personalized molecularly targeted therapies have demonstrated limited success. However, results of large studies such as the National Cancer Institute's Molecular Analysis for Therapy Choice (NCI-MATCH) and the American Society of Clinical Oncology's Targeted Agent and Profiling Utilization Registry (TAPUR) will offer more definitive results.

As sarcoma is not a homogenous malignancy but a conglomerate of over 50 unique histologies, achieving adequate sample sizes to conduct molecular profiling studies within specific subtypes is challenging. Molecular profiling of more common, genetically complex sarcoma histologies has been reported, including liposarcoma, leiomyosarcoma, and osteosarcoma with varied findings. For example, genomic evaluation of 86 patients with liposarcoma identified amplification of specific genes on chromosome 12q (previously known to be amplified) as well as recurrent mutations in 7 genes, many with downstream pathways that could potentially be targeted [10]. A similar study of 54 patients with leiomyosarcoma revealed recurrent mutations in *TP53* and *ATRX* genes [11]. The clinical benefit of these findings for patients is unclear, although the data supports the heterogeneity of sarcomas observed clinically.

In this study, we analyzed tumors of six EMC patients to identify actionable molecular target(s) and improve understanding of disease biology. Similar to other translocation-associated sarcomas, the mutational profile of EMC was limited beyond the pathognomonic translocation. While other genomic aberrations were found, none were recurrent or considered clinically relevant. Potential explanations include the limited sample size or that a specific class of molecular aberrations in EMC, such as aberrant methylation signatures (not a component of the MI-ONCOSEQ integrative analysis), has not been identified.

It is also possible that in translocation-associated sarcomas, the translocation alone is sufficient for both initiation and progression of disease. This idea is supported by Agaram et al who showed a correlation with fusion type and clinical behavior. Specifically, EMCs with variant *NR4A3* gene fusions demonstrate a clinically more aggressive phenotype than EMCs with the classic *EWSR1-NR4A3* fusion. Their analysis of 26 EMCs found *EWSR1-NR4A3*, *TAF15-NR4A3*, and *TCF12-NR4A3* in 62%, 27%, and 4%, respectively. EMCs with variant fusions had increased cellularity, proliferation, and atypia including plasmacytoid/rhabdoid morphology in 50% of cases. These patients also had worse survival outcomes compared with EMC patients with the *EWSR1-NR4A3* fusion [12]. The finding of a more aggressive phenotype with the variant fusion corroborates the previously mentioned study of sunitinib in EMC. The two patients with the variant fusion, *TAF15-NR4A3*, had progressive disease on sunitinib, while all patients with the classical translocation had stable or responsive disease [7]. Given that our study did not include patients with variant fusions, there may be genetic aberrations in these EMCs that we did not identify. EMC is also a disease with a male predilection for unknown reason. Evaluation comparing genetic abnormalities in male and female specimens may offer insight.

EMC is a malignancy that frequently becomes metastatic, and clearly effective therapy to delay progression and improve survival has not been identified. A novel molecular target was not identified in these samples. However, we observed overexpression of sunitinib target genes, specifically *RET*, consistent with recent reports of *RET* expression observed in EMC patients treated with this multi-kinase inhibitor. Although multi-kinase inhibitors are approved and widely used for malignancies such as renal cell carcinoma (pazopanib, sorafenib, sunitinib), soft tissue sarcoma (pazopanib), and hepatocellular carcinoma (sorafenib), there has not been a well-described, consistent predictive biomarker of drug response. Analysis of a larger series of EMC patients may identify predictive biomarkers, but the clinical benefit of sunitinib in EMC needs confirmation in a randomized clinical trial.

Future treatment strategies for EMC would ideally consist of targeting the pathognomonic fusion transcription factor or the downstream effectors of the translocation partner *NR4A3*, a member of the steroid-thyroid hormone-retinoid receptor superfamily that activates peroxisome proliferator-activated receptor-gamma (PPAR-gamma). In our EMC cohort, one patient with diabetes mellitus was treated with an antidiabetic agent, pioglitazone, a thiazolidinedione that is a potent and selective agonist for PPAR-gamma. The patient had stable metastatic EMC for 13 months before unequivocal progression. The contribution of pioglitazone to disease control is unknown. The folate receptor (FOLR1) is known to be overexpressed in many tumor types including ovarian, breast, and lung [13], and this was also found in two of our EMC samples. Given the availability of antifolate agents, this could be a potential therapeutic strategy for EMC.

In summary, next generation sequencing did not reveal clinically actionable targets in our cohort of EMC patients, although the sample size in our study is relatively small. Given the rarity of EMC, combining clinical sequencing efforts of additional EMC patients in a national or international collaboration may be the most informative approach. Additional pre-clinical investigations of EMC using patient-derived xenograft or organoid culture experimental models may help elucidate the molecular mechanisms of EMC tumorigenesis that could be translated to the clinical setting, particularly understanding the effect of *RET* expression as *RET* is clinically targetable.

## PATIENTS AND METHODS

Between January 31, 2012 and April 15, 2016, five patients with EMC underwent tumor biopsy of a metastatic site and previously fresh-frozen metastatic tumor tissue from one patient was submitted as part of an integrative clinical sequencing research study, MI-ONCOSEQ, at the University of Michigan. Somatic tissue from buccal swabs and/or peripheral blood was also collected and submitted for analysis paired to tumor tissue. The study was approved by the institutional Internal Review Board (Michigan Oncology Sequencing Protocol, IRB# HUM00046018). Informed consent was obtained from all patients prior to biopsy detailing risks of integrative sequencing and included genetic counseling to discuss the potential of incidental germline findings. Eligibility criteria for the study include medically fit patients over the age of 18 with advanced or refractory sarcoma. The detailed findings in patients with EMC are detailed herein.

Needle biopsies were snap frozen in OCT, and longitudinal sections were cut. Hematoxylin and eosin (H&E)-stained frozen sections were reviewed by the study pathologist (L.P.K.) to identify cores with the highest tumor content that were subsequently used for nucleic acid extraction.

High quality DNA and RNA was isolated from the core needle biopsies and matched normal tissue. Samples were processed to undergo comprehensive next generation sequencing (whole exome (OncoSeq capture exome for patient MO_1582) of tumor and normal, tumor PolyA+ and capture transcriptome). Briefly, exome libraries of tumor and matched normal genomic DNAs were generated using the Illumina TruSeq DNA Sample Prep Kit, following the manufacturer's instructions. RNA-Seq transcriptome libraries were prepared following Illumina's TruSeq RNA protocol, using 2ug of total RNA. RNA integrity was measured using an Agilent 2100 Bioanalyzer. The quality and quantity of the resulting exome and transcriptome libraries were analyzed using an Agilent 2100 Bioanalyzer and DNA 1000 reagents. Paired-end libraries were sequenced with the Illumina HiSeq 2500. Reads that passed the chastity filter of Illumina BaseCall software were used for subsequent analysis. MI-ONCOSEQ bioinformatic pipelines are used to detect the following classes of mutations: somatic and germline variant calls including single-nucleotide variants and insertion/deletions (indels), copy number alterations, gene fusions and outlier gene expression. Potentially actionable mutations are defined as any genomic findings discovered during sequencing that could lead to a (1) change in patient management by providing a targetable molecular aberration; (2) change in diagnosis or risk stratification and/or; (3) cancer-related germline findings that inform about a potential cancer risk to patient family members.

The medical records of all patients were examined to obtain relevant clinical data. Findings for each patient were presented and discussed at an institutionally sanctioned Precision Medicine Tumor Board (PMTB). PMTB occurs biweekly and includes pathologists, adult and pediatric medical oncologists, biologists, bioinformaticians, geneticists, genetic counselors, bioethicists, study coordinators. Results of the PMTB are discussed with patients by their medical oncologists along with geneticists and genetics counselors when needed.

## SUPPLEMENTARY MATERIALS FIGURES AND TABLES




