# Supplementary material for: Next generation sequencing of extraskeletal myxoid chondrosarcoma
Source: Oncotarget. 2017 Feb 21;8(13):21770–7. doi: 10.18632/oncotarget.15568 (PMC5400622; doi:10.18632/oncotarget.15568)
Supplement: Supplementary file 1 [file oncotarget-08-21770-s001.pdf]

www.impactjournals.com/oncotarget/ Oncotarget, Supplementary Materials 2016

Next generation sequencing of extraskeletal myxoid chondrosarcoma

SUPPLEMENTARY FILE

See Supplementary File 1
