# Supplementary material for: Next generation sequencing of extraskeletal myxoid chondrosarcoma
Source: Oncotarget. 2017 Feb 21;8(13):21770–7. doi: 10.18632/oncotarget.15568 (PMC5400622; doi:10.18632/oncotarget.15568)
Supplement: Supplementary file 2 [file oncotarget-08-21770-s002.docx]

**MO_1023: PATIENT HISTORY**

54 year-old man with metastatic extraskeletal myxoid chondrosarcoma. In January 2009, he developed cramp-like feeling in his left calf with noticeable edema. He underwent lower extremity venous duplex scanning, which revealed extensive acute DVT. Ultrasound also revealed an approximately 5 x 4 centimeter mass in the proximal thigh/left groin. The mass appeared to be compressing the proximal portion of the superficial femoral vein.

Additional evaluation included MRI of the thigh, which revealed 14 x 14 centimeter mass. CT scan of chest, abdomen, and pelvis, revealed multiple bilateral pulmonary nodules measuring about 5 millimeters in size. Chest CT revealed incidental finding of a pulmonary embolus in the left main pulmonary artery and multiple smaller peripheral emboli, bilaterally. He was started on anticoagulation with warfarin. CT- guided fine-needle aspiration of the mass suggested extraskeletal myxoid chondrosarcoma. FISH testing revealed a translocation involving the EWSR1 gene (22 q 1.2).

He underwent resection of his large left thigh mass on April 15, 2009. In October 2009, there was definite but small growth of lung nodules, and he was asymptomatic. He opted for laser treatment and resection of lung nodules, November 2010. The largest nodules were resected, although the left lung was not completely cleared of nodules. Again, in October 2011, he had multiple laser treatment and resection of nodules from his right lung.

He has been observed since then. Based on his last CT scan from October 2011, he does have some residual disease in his left lung.

**MO_1023 Sequencing libraries**

| **Study ID** | **Tissue Block** | **Tissue size** | **Tumor content** | **Sample type** | **Yield** | **RNA RIN** | **Amount for Lib prep** | **Lib type** | **Exome platform** | **Library ID** | **Barcode** |
| --- | --- | --- | --- | --- | --- | --- | --- | --- | --- | --- | --- |
| MO_1023  Tumor | 3 | 1.4cm (half) | 60% | RNA | 7.5ug | 2.7 | 2 ug | Poly(A) Transcriptome | - | SI_4828 | 3 |
| MO_1023  Tumor | 3 | 1.4cm (half) | 60% | RNA | 7.5ug | 2.7 | 2 ug | Transcriptome- Exome capture | Roche Ez Exome v2 | SI_4824 | 11 |
| MO_1023  Tumor | 3 | 1.4cm  (half) | 60% | DNA | 10ug | NA | 3 ug | Exome | Roche Ez  Exome v2 | SI_4819 | 6 |
| MO_1023  Normal | Blood (Javed) |  | 0% | DNA | 45ug | NA | 3 ug | Exome | Roche Ez Exome v2 | SI_4818 | 5 |
| MO_1023  Tumor FFPE | F 04 | 1.8x1.4  cm | 80% | RNA | 7.5ug | 2.7 | 3 ug | Transcriptome- Exome capture | Agilent V4 Oligos | SI_4973 | 11 |
| MO_1023  Tumor FFPE | F 04 | 1.8x1.4  cm | 80% | DNA | 0.3ug | NA | 0.3 ug | Exome | Agilent V4 Oligos | SI_4991 | 2 |
| MO_1023  Normal | Blood (Javed) |  | 0% | DNA | 45 ug | NA | 3 ug | Exome | Agilent V4 Oligos | SI_5038 | 5 |

*Dan Robinson, Yi-Mi Wu, Xuhong Cao, Sequencing group*

**MO_1023 SNP Fingerprinting Analysis**

|  | SI_4818  (Normal exome) | SI_4819  (Tumor exome) | SI_4828  (Tumor transcriptome) | SI_4973  (Tumor transcriptome) | SI_4991  (Tumor exome) | SI_5038  (Normal exome) |
| --- | --- | --- | --- | --- | --- | --- |
| SI_4818  (Normal exome) | 135/135 (100%) | 135/135 (100%) | 9/9 (100%) | 121/122 (99%) | 127/127 (100%) | 119/119 (100%) |
| SI_4819  (Tumor exome) | 135/135 (100%) | 146/146 (100%) | 10/10 (100%) | 130/131 (99%) | 137/137 (100%) | 129/129 (100%) |
| SI_4828  (Tumor transcriptome) | 9/9 (100%) | 10/10 (100%) | 10/10 (100%) | 8/8 (100%) | 9/9 (100%) | 9/9 (100%) |
| SI_4973  (Tumor transcriptome) | 121/122 (99%) | 130/131 (99%) | 8/8 (100%) | 135/135 (100%) | 125/126 (99%) | 120/121 (99%) |
| SI_4991  (Tumor exome) | 127/127 (100%) | 137/137 (100%) | 9/9 (100%) | 125/126 (99%) | 139/139 (100%) | 130/130 (100%) |
| SI_5038  (Normal exome) | 119/119 (100%) | 129/129 (100%) | 9/9 (100%) | 120/121 (99%) | 130/130 (100%) | 132/132 (100%) |


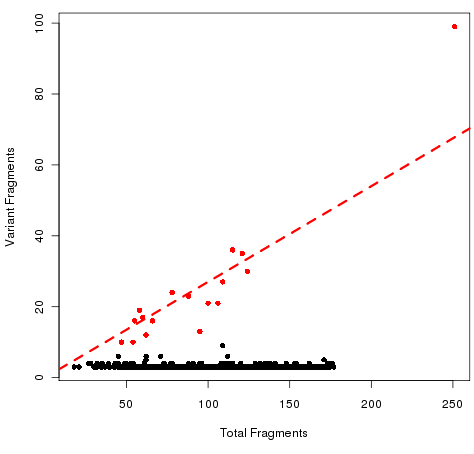
**MO_1023 Estimated Tumor Content = 54% (Frozen tissue)**

**MO_1023 Estimated Tumor Content = 14% (FFPE tissue)**


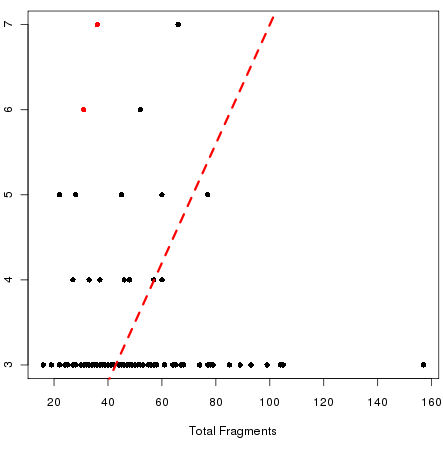


*Bob Lonigro*

**MO_1023 Copy Number Profile**


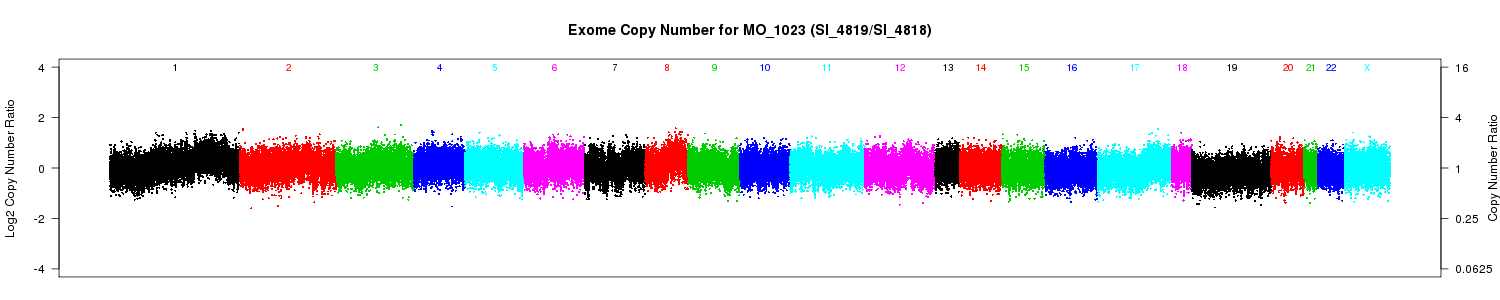


**Frozen tissue**


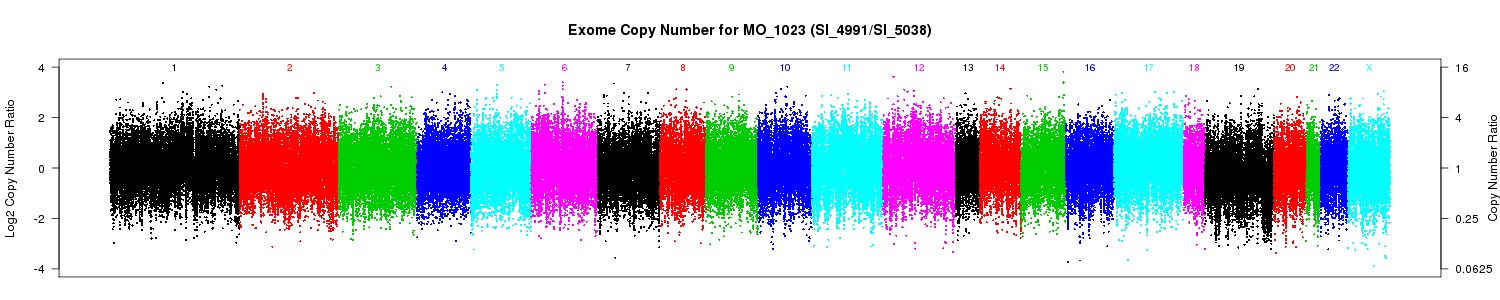


**FFPE tissue**

*Bob Lonigro*

**MO_1023: Total 19 Somatic mutations (Frozen tissue)**

| **Patient** | **Gene** | **SNV Location** | **Base change** | **Tumor Variant Fraction** | **Protein** | **BLOSUM**  **Score** | **COSMIC**  **Mutations** | **MO_1039**  **Gene Expression (RPKM)** | **Median Gene Expression across Compendium Tissues (RPKM)** | **Copy Number Ratio** |
| --- | --- | --- | --- | --- | --- | --- | --- | --- | --- | --- |
| MO_1023 | BANF1 | chr11:65527764 | A→G | 23/88 (26%) | p.K72R | 2 | 0/90 | 56.39 | 27.6 | 0.9 |
| MO_1023 | PALLD | chr4:169669901 | T→C | 7/62 (11%) | p.M224T | -1 | 3/96 | 12.05 | 12.84 | 1.09 |
| MO_1023 | NES | chr1:154907302 | G→A | 12/15 (80%) | p.P1101L | -3 | 6/520 | 9.52 | 8.67 | 1.07 |
| MO_1023 | MMAB | chr12:108495613 | C→T | 12/23 (52%) | p.R19H | 0 | 0/90 | 5.27 | 7.76 | 0.94 |
| MO_1023 | OLFML1 | chr11:7487841 | G→A | 16/55 (29%) | p.S352N | 1 | 3/71 | 3.23 | 1.99 | 1.04 |
| MO_1023 | ZC3H3 | chr8:144691485 | T→C | 14/29 (48%) | p.S399G | 0 | 3/93 | 1.26 | 8.07 | 1.18 |
| MO_1023 | ICAM5 | chr19:10263938 | G→A | 9/17 (53%) | p.V301I | 3 | 3/153 | 1.14 | 0.2 | 0.79 |
| MO_1023 | PAK2 | chr3:197993974 | C→G | 11/64 (17%) | p.S20R | -1 | 2/948 | 1.06 | 7.52 | 0.78 |
| MO_1023 | IFT140 | chr16:1574386 | C→T | 7/18 (39%) | p.V398I | 3 | 7/99 | 0.41 | 6.19 | 0.88 |
| MO_1023 | CROCC | chr1:17137507 | C→T | 8/34 (24%) | p.A439V | 0 | 6/138 | 0.29 | 4.7 | 0.92 |
| MO_1023 | PSPH | chr7:56056319 | T→A | 9/53 (17%) | p.R27S | -1 | 2/92 | 0.27 | 2.7 | 0.73 |
| MO_1023 | TBCK | chr4:107334296 | C→T | 36/115 (31%) | p.V597I | 3 | 4/326 | 0.02 | 5.12 | 1.08 |
| MO_1023 | FBN3 | chr19:8082640 | C→T | 8/15 (53%) | p.V1326I | 3 | 17/105 | 0.01 | 0.1 | 0.82 |
| MO_1023 | DSG1 | chr18:27167598 | G→A | 30/124 (24%) | p.G245R | -2 | 9/521 | 0 | 0.01 | 1.04 |
| MO_1023 | UBQLNL | chr11:5493221 | T→C | 99/251 (39%) | p.T343A | 0 | 4/137 | 0 | 0.37 | 1 |
| MO_1023 | FIG4 | chr6:110219379 | G→A | 12/62 (19%) | p.G763E | -2 | 2/70 | 0 | 6.07 | 1.04 |
| MO_1023 | OR51B6 | chr11:5329671 | T→C | 9/81 (11%) | p.C120R | -3 | 2/70 | 0 | 0 | 1 |
| MO_1023 | WDR65 | chr1:43445023 | G→A | 21/106 (20%) | p.G530S | 0 | 2/92 | 0 | 0.12 | 0.92 |
| MO_1023 | DTX2 | chr7:75969581 | A→G | 8/26 (31%) | p.T374A, p.T421A | 0 | 2/524 | 0 | 4.48 | 0.76 |

**MO_1023: Total 8 Somatic mutations (FFPE tissue)**

| **Patient** | **Gene** | **SNV Location** | **Base change** | **Tumor Variant Fraction** | **Protein** | **BLOSUM**  **Score** | **COSMIC**  **Mutations** | **Copy Number Ratio** |
| --- | --- | --- | --- | --- | --- | --- | --- | --- |
| MO_1023 | BANF1 | chr11:65527764 | A→G | 17/34 (50%) | p.K72R | 2 | 0/90 | 1.67 |
| MO_1023 | MCF2L | chr13:112781044 | A→G | 6/16 (38%) | p.I657V, p.I598V, p.I604V | 3 | 2/219 | 0.68 |
| MO_1023 | TBCK | chr4:107334296 | C→T | 13/20 (65%) | p.V597I | 3 | 4/326 | 1.22 |
| MO_1023 | TMEM211 | chr22:23664114 | A→C | 7/25 (28%) |  |  | 1/69 | 0.69 |
| MO_1023 | RAI1 | chr17:17637821 | G→C | 7/63 (11%) | p.Q278H | 0 | 3/92 | 1.83 |
| MO_1023 | SEMG1 | chr20:43270692 | G→A | 6/19 (32%) | p.R447H | 0 | 2/92 | 0.6 |
| MO_1023 | FIG4 | chr6:110219379 | G→A | 16/30 (53%) | p.G763E | -2 | 2/70 | 0.83 |
| MO_1023 | XPC | chr3:14174913 | T→C | 14/49 (29%) | p.I492V, p.I455V | 3 | 5/655 | 1.78 |

**MO_1023 Fusion Candidates (FFPE tissue)**

| **Patient** | **Library** | **Genes (5',3')** | **Reads** | **Type** | **Comments** | **Copy Number Ratio** |
| --- | --- | --- | --- | --- | --- | --- |
| MO_1023 | SI_4973 | EWSR1-NR4A3 | 1196 | Inter | extraskeletal myxoid chondrosarcoma, but not detected in previous run - D0L3RACXX_4824 & 4828 | 1.23, 1.00 |

*Shanker Kalyana-Sundaram*

Patient is a 66 y.o. male with a history of diabetes and hypertension with a left deltoid mass noticed in May 2011. Biopsy of the deltoid mass showed an extraskeletal myxoid chondrosarcoma; he also had multiple lung nodules at diagnosis. Given the lack of chemotherapy and radiation sensitivity of this pathology, recommendation was for palliative resection of his primary deltoid malignancy.

**MO_1088: PATIENT HISTORY**

Patient resection of his left shoulder sarcoma with pathology showing an 8.5 centimeter low- grade extraskeletal myxoid chondrosarcoma with nearly free margins. He has been in follow- up since that time with periodic imaging.

10/24/12 Chest CT results: Innumerable pulmonary metastatic nodules, several of which have increased in size - approximate increase in size of 40%. No new pulmonary nodules. Findings are concerning for progressive disease.

Patient underwent a lung biopsy on 11/20/12 for MI-ONCOSEQ.

# Libraries

**MO_1088: SEQUENCING SAMPLES**

| **Study ID** | **Tissue Block** | **Specimen size** | **Tumor content** | **Sample type** | **Yield** | **RNA RIN** | **Amount for Lib prep** | **Lib type** |
| --- | --- | --- | --- | --- | --- | --- | --- | --- |
| MO_1088  Tumor | 2+4 | 0.1+0.1cm | 40-60% | RNA | 0.5ug | NA | 0.5ug | Exome Cap- Transcriptome |
| MO_1088  Tumor | 5+6 | 0.1+0.1cm | 50-80% | DNA | 1.5ug | NA | 1.4ug | Exome |
| MO_1088  Normal | Blood (Javed) | - | 0% | DNA | 50ug | NA | 3ug | Exome |

Tumor and normal exome and capture RNA libraries were analyzed.

***SNP Fingerprinting Analysis***

**MO_1088: SEQUENCING SAMPLES- QC**

|  | **SI_5716**  **(Normal Exome)** | **SI_5831**  **(E.C. Transcriptome)** | **SI_5715**  **(Tumor Exome)** |
| --- | --- | --- | --- |
| **SI_5716 (Normal Exome)** | 159/159 (100%) | 159/159 (100%) | 159/159 (100%) |
| **SI_5831 (E.C.**  **Transcriptome)** | 159/159 (100%) | 166/166 (100%) | 161/161 (100%) |
| **SI_5715 (Tumor Exome)** | 159/159 (100%) | 161/161 (100%) | 161/161 (100%) |

Genotype analysis reveals 100% identity match within patient samples.

***SNP Fingerprinting Analysis***

**MO_1088: SEQUENCING SAMPLES- QC**

(Comparison To Other Patients)

| **Other Patient's Library** | **MO_1088's Library** | **Concordant SNPs** | **Total SNPs** | **Concordanc e** |
| --- | --- | --- | --- | --- |
| SI_5503 (MO_1078 Tumor Exome Capture Transcriptome ) | SI_5831 ( Tumor Exome Capture Transcriptome ) | 103 | 164 | 62.8% |
| SI_5556 (MO_1085 Tumor Exome Capture Transcriptome ) | SI_5716 ( Normal Exome ) | 99 | 158 | 62.7% |
| SI_5503 (MO_1078 Tumor Exome Capture Transcriptome ) | SI_5715 ( Tumor Exome ) | 101 | 161 | 62.7% |
| SI_5503 (MO_1078 Tumor Exome Capture Transcriptome ) | SI_5716 ( Normal Exome ) | 99 | 159 | 62.3% |
| SI_5552 (MO_1085 Tumor Transcriptome ) | SI_5716 ( Normal Exome ) | 97 | 156 | 62.2% |
| SI_5556 (MO_1085 Tumor Exome Capture Transcriptome ) | SI_5715 ( Tumor Exome ) | 99 | 160 | 61.9% |
| SI_5568 (MO_1085 Normal Exome ) | SI_5716 ( Normal Exome ) | 97 | 157 | 61.8% |
| SI_5567 (MO_1085 Tumor Exome ) | SI_5716 ( Normal Exome ) | 97 | 157 | 61.8% |
| SI_5552 (MO_1085 Tumor Transcriptome ) | SI_5715 ( Tumor Exome ) | 97 | 158 | 61.4% |
| SI_5556 (MO_1085 Tumor Exome Capture Transcriptome ) | SI_5831 ( Tumor Exome Capture Transcriptome ) | 101 | 165 | 61.2% |
| SI_5568 (MO_1085 Normal Exome ) | SI_5831 ( Tumor Exome Capture Transcriptome ) | 97 | 159 | 61.0% |
| SI_5568 (MO_1085 Normal Exome ) | SI_5715 ( Tumor Exome ) | 97 | 159 | 61.0% |
| SI_5567 (MO_1085 Tumor Exome ) | SI_5715 ( Tumor Exome ) | 97 | 159 | 61.0% |
| SI_5552 (MO_1085 Tumor Transcriptome ) | SI_5831 ( Tumor Exome Capture Transcriptome ) | 99 | 163 | 60.7% |
| SI_5567 (MO_1085 Tumor Exome ) | SI_5831 ( Tumor Exome Capture Transcriptome ) | 97 | 160 | 60.6% |
| SI_5487 (PO_3009 Tumor Transcriptome ) | SI_5715 ( Tumor Exome ) | 96 | 159 | 60.4% |

Patient genotype is distinguishable from all other patients, ie: closest match of 62.8% with MO_1078.


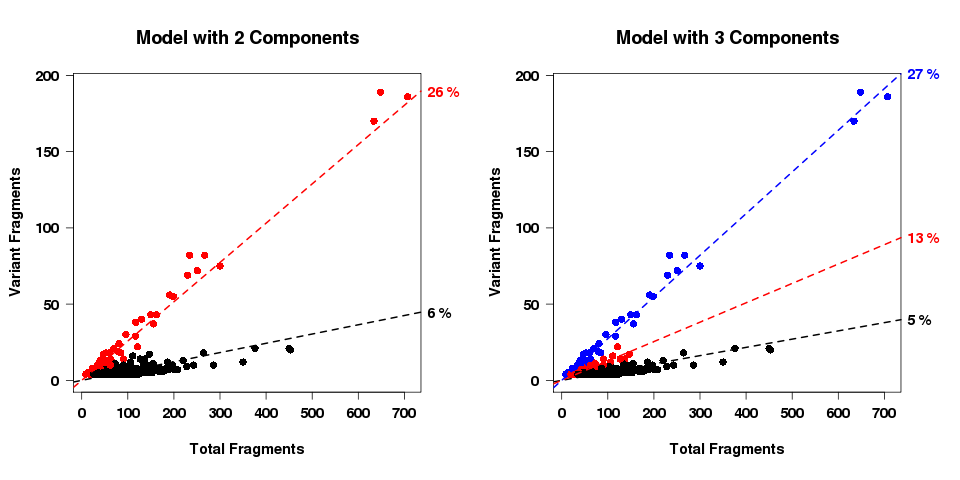
***Tumor Content***

**MO_1088: SEQUENCING SAMPLES- QC**

**Estimated Tumor Content = 52%**

***Summary***

**MO_1088: SEQUENCING SAMPLES- QC**

| **Identity:** | √ |
| --- | --- |
| **RNA:** | RIN = NA |
| **Transcriptome:** | Capture only (small tissues, low RNA quantity) |
| **Exome:** | Tumor vs. Normal |
| **Tumor content:** | 52% |


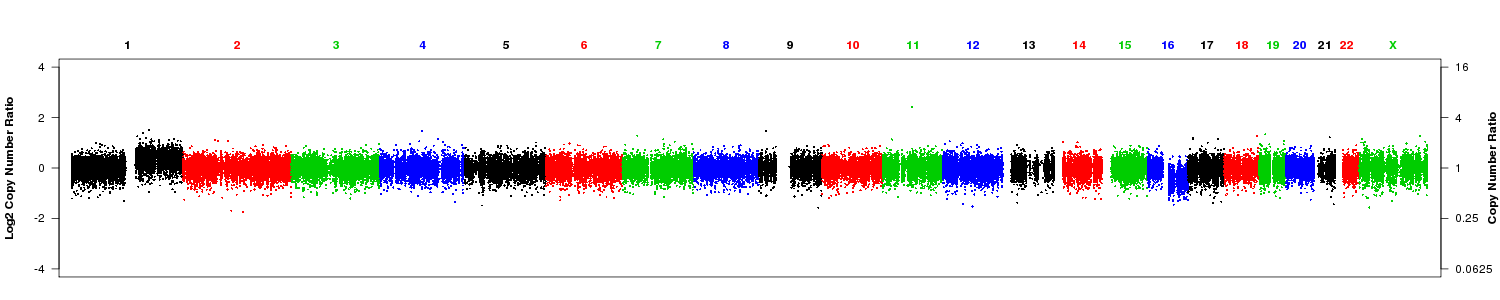
***Copy Number Profile***

**MO_1088: SEQUENCE ANALYSIS SUMMARY**

**1q gain 16q loss**


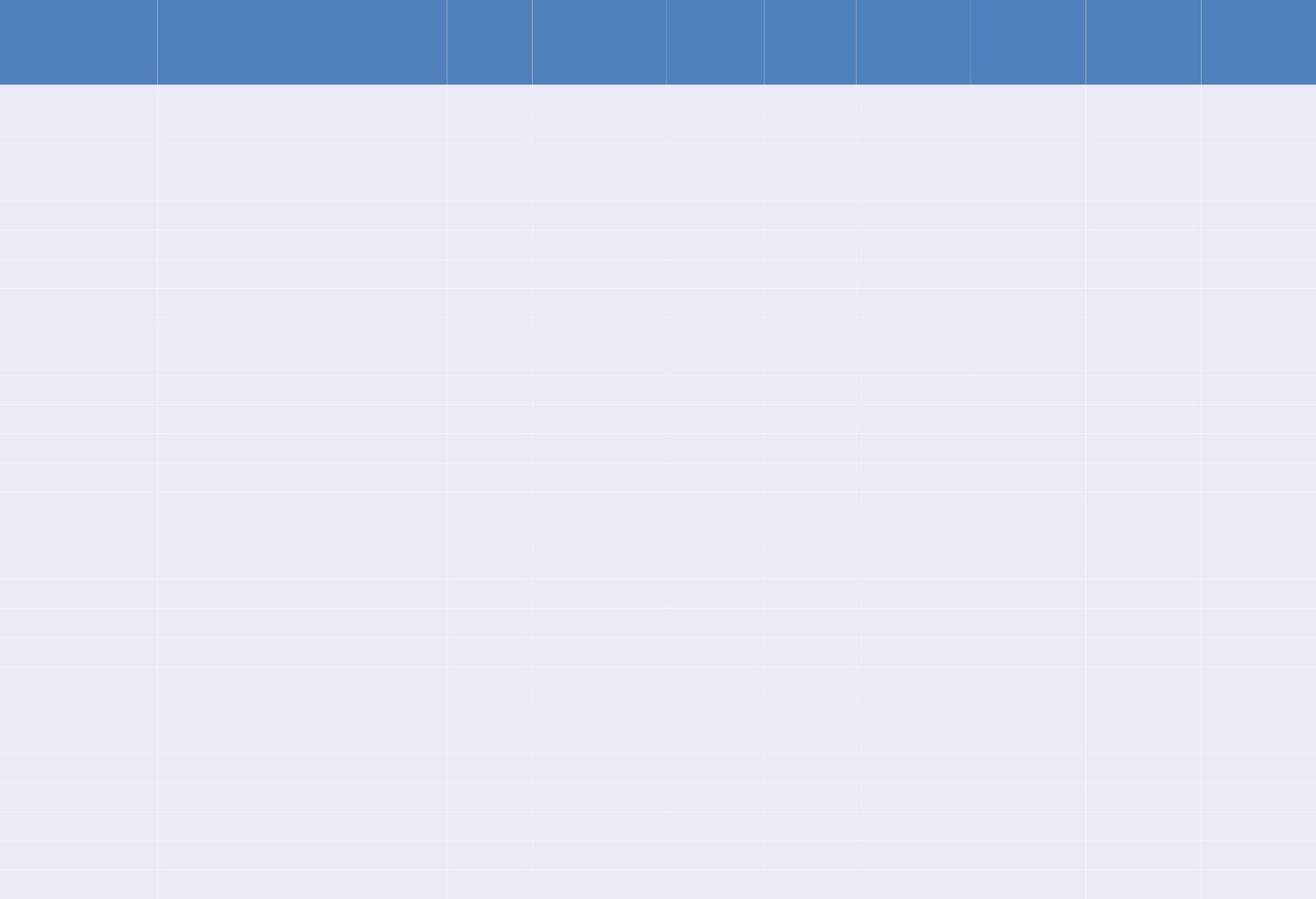
***Total somatic mutations: 28***

**MO_1088: SEQUENCE ANALYSIS SUMMARY**

| **Gene Name** | **Amino Acid Change** | **Chr** | **Coord** | **Ref** | **Var** | **Tumor Read Depth** | **Ref Reads**  **> Q20**  **Tumor** | **Var Reads**  **> Q20**  **Tumor** | **Var Allele**  **Freq %** |
| --- | --- | --- | --- | --- | --- | --- | --- | --- | --- |
| AC010336.1 | p.P751S | 19 | 7935879 | G | A | 62 | 45 | 12 | 21.1 |
| ARSD | p.G221S | X | 2836047 | C | T | 62 | 52 | 9 | 14.8 |
| ARSD | p.F223I | X | 2836041 | A | T | 60 | 52 | 8 | 13.3 |
| BRIP1 | p.V607A | 17 | 59857737 | A | G | 55 | 36 | 18 | 33.3 |
| C9orf69 | p.R27W | 9 | 139008742 | G | A | 121 | 89 | 29 | 24.6 |
| CACNA1S | p.R1580Q | 1 | 201013457 | C | T | 718 | 566 | 128 | 18.4 |
| CBLC | p.R118K | 19 | 45281541 | G | A | 36 | 28 | 7 | 20.0 |
| CES3 | p.A66P | 16 | 66997195 | G | C | 264 | 127 | 21 | 14.2 |
| CIB2 | p.R66W | 15 | 78403509 | G | A | 132 | 91 | 40 | 30.5 |
| CPZ | p.P6L | 4 | 8594577 | C | T | 31 | 17 | 7 | 29.2 |
| CPZ | p.L5P | 4 | 8594574 | T | C | 29 | 16 | 6 | 27.3 |
| ERBB4 | p.E133D (not hot spot) | 2 | 212812177 | T | A | 83 | 58 | 24 | 29.3 |
| GALR3 | p.T56P | 22 | 38219579 | A | C | 591 | 327 | 51 | 13.5 |
| MKL1 | p.A567T,p.A517T | 22 | 40814743 | C | T | 15 | 8 | 7 | 46.7 |
| MST1P9 | p.A583V,p.A609V | 1 | 17084269 | G | A | 33 | 27 | 6 | 18.2 |
| NCDN | p.A714P,p.A697P | 1 | 36031214 | G | C | 125 | 70 | 8 | 10.3 |
| NLGN3 | p.N126K | X | 70367977 | C | G | 346 | 147 | 181 | 55.2 |
| PCDHGA6 | p.P500L | 5 | 140755149 | C | T | 737 | 522 | 186 | 26.3 |
| POTEG | p.A146T | 14 | 19553852 | G | A | 49 | 40 | 5 | 11.1 |
| PTPN21 | p.Y569H | 14 | 88946070 | A | G | 242 | 145 | 16 | 9.9 |
| PTPRA | p.G656V (not hot spot) | 20 | 3016277 | G | T | 311 | 226 | 75 | 24.9 |
| RASA2 | p.C779F,p.C780F | 3 | 141328725 | G | T | 43 | 25 | 5 | 16.7 |
| SERPINB7 | p.R249X,p.R266X | 18 | 61471522 | C | T | 56 | 40 | 13 | 24.5 |
| SERPINC1 | p.S426L | 1 | 173873145 | G | A | 173 | 131 | 39 | 22.9 |
| SIK1 | p.T479P | 21 | 44838928 | T | G | 175 | 46 | 5 | 9.8 |
| TPSAB1 | p.R168P | 16 | 1291831 | G | C | 18 | 12 | 6 | 33.3 |
| ZDBF2 | p.A1090E | 2 | 207172521 | C | A | 156 | 108 | 43 | 28.5 |
| ZNF573 | p.M535I | 19 | 38229612 | C | A | 125 | 100 | 22 | 18.0 |

***Fusion candidates***

**MO_1088: SEQUENCE ANALYSIS SUMMARY**

| **5' Gene** | **5' Chr** | **5' Coord** | **3' Gene** | **3' Chr** | **3' Coord** | **Spanning Reads** | **Spanning Mate Pairs** | **Spanning Mate Pairs w/ Fusion** |
| --- | --- | --- | --- | --- | --- | --- | --- | --- |
| **EWSR1** | 9 | 102590322 | **NR4A3** | 22 | 29692357 | 800 | 254 | 928 |
| **EWSR1** | 9 | 102590364 | **NR4A3** | 22 | 29692357 | 42 | 254 | 50 |
| **EWSR1** | 9 | 102589011 | **NR4A3** | 22 | 29692357 | 33 | 254 | 40 |
| DNHD1 | 11 | 6520168 | RRP8 | 11 | 6624633 | 33 | 5 | 35 |
| SAMD5 | 6 | 147830522 | SASH1 | 6 | 148711269 | 9 | 1 | 8 |
| DAD1 | 14 | 23043958 | AE000661.37.1 | 14 | 22872163 | 5 | 1 | 6 |
| EIF4E3 | 3 | 71739160 | FOXP1 | 3 | 71542705 | 4 | 2 | 3 |
| LOC728723 | 5 | 76414345 | PDE8B | 5 | 76607818 | 3 | 2 | 3 |
| SLC14A1 | 18 | 43327241 | EPG5 | 18 | 43435537 | 4 | 2 | 3 |
| FAM108B1 | 9 | 74477466 | TMEM2 | 9 | 74365300 | 2 | 1 | 2 |

***Germline Variations***

**MO_1088: SEQUENCE ANALYSIS SUMMARY**

| **Gene Name** | **Amino Acid Change** | **Chr** | **Coord** | **Ref** | **Var** | **Tumor Read Depth** | **Ref Reads**  **> Q20**  **Tumor** | **Var Reads**  **> Q20**  **Tumor** | **Var Allele Freq %** | **1000**  **Genome** | **AVISIFT**  **Score** |
| --- | --- | --- | --- | --- | --- | --- | --- | --- | --- | --- | --- |
| RNASEL | p.I97L | 1 | 182555653 | T | G | 801 | 492 | 277 | 36.0 | 0.01 | 0.17 |
| ALK | p.H1030P | 2 | 29448410 | T | G | 306 | 114 | 34 | 23.0 |  | 0.26 |
| FANCD2 | p.G901V | 3 | 10115033 | G | T | 78 | 46 | 30 | 39.5 | 0.01 | 0.05 |
| COL7A1 | p.V198I | 3 | 48630625 | C | T | 342 | 161 | 165 | 50.6 |  | 0.24 |
| SBDS | p.I212T | 7 | 66453476 | A | G | 130 | 64 | 59 | 48.0 | 0.02 | 0.05 |
| BLM | p.P868L | 15 | 91326099 | C | T | 34 | 21 | 12 | 36.4 | 0.05 | 0 |
| PALB2 | p.G1084V | 16 | 23619284 | G | A | 48 | 21 | 22 | 51.2 |  | 0.27 |
| BRCA1 | p.Q60R,p.Q330 R,p.Q309R,p.Q 356R | 17 | 41246481 | T | C | 149 | 71 | 71 | 50.0 | 0.03 | 0.03 |

**MO_1088: SEQUENCE ANALYSIS SUMMARY**

| **Mutation class** | **Gene/Aberration** | **Potential Therapies/*Clinical Trials**  **(*Contingent on meeting study eligibility criteria )** |
| --- | --- | --- |
| Copy number variation | 1q gain 16q loss |  |
| Somatic point mutations (Total:28) | Detected, unknown significance |  |
| Insertions/deletions (indels) | N/A |  |
| Gene fusions | EWSR1-NR4A3 |  |
| Outlier expression | N/A |  |
| Germline variants | N/A |  |
| Pathogens | N/A |  |

**PRECISION TUMOR BOARD DISCUSSION/INTERPRETATION**

- *Aberrations that may relate to standard of care:* N/A
- *Aberrations that may make patient eligible for an open clinical trial or other therapies:*

The EWSR1-NR4A3 activates PPAR-gamma (*J Pathol*. 2009 Jan;217(1):83-93);agonists of PPAR- gamma are known to trigger differentiation. A number of agonists have been developed to treat diabetes but use was largely discontinued due to adverse side effects. Any currently available compounds may be considered for off-label use for this patient.

- *Germline mutations/family history- implications for disclosure:* N/A
- *Other informative results:*

- Trisomy 1q is frequently observed in extraskeletal myxoid chondrosarcoma (Am J Pathol. 2003 Mar; 162(3):781-92).

**MO_1180: PATIENT HISTORY**

Patient is a 58-year-old male with extraskeletal myxoid chondrosarcoma.

The patient first noticed pain in the right lateral aspect of his calf in June/July of 2011. He felt a lump in his right hip around October/November of 2011 after falling. Imaging performed on 12/8/2011 revealed a lesion with scalloped margins. Further imaging done on 2/7/2012 revealed a lytic lesion at the right inferior pubic ramus, a mass within the right thigh musculature, and scattered pulmonary nodules in the chest. A biopsy performed on 2/7/2012 of the right ischial bone and the soft tissue mass in the hip were read as myxoid sarcoma.

Follow-up imaging demonstrated disease progression, so he received palliative radiation to the base of the skull, the buttock, and the left hilum from 3/25/13 until 4/10/13. He completed radiation to the right humerus on 5/8/13. Imaging performed on 6/10/13 revealed extensive progression of disease.

The patient underwent a left gluteal mass biopsy for MI-ONCOSEQ.

**MO_1180 Sequencing libraries**

| **Study ID** | **Tissue Block** | **Specimen size** | **Tumor content** | **Sample type** | **Yield** | **RNA RIN** | **Amount for Lib prep** | **Lib type** | **Exome platform** | **Library ID** | **Barcode** |
| --- | --- | --- | --- | --- | --- | --- | --- | --- | --- | --- | --- |
| MO_1180  Tumor | 2+4+5 | 0.3+0.3+0.1  cm | 50-65% | RNA | 2.4ug | 9 | 1.2ug | Poly(A)+ Transcriptome | - | SI_6691 | 4 |
| MO_1180  Tumor | 2+4+5 | 0.3+0.3+0.1  cm | 50-65% | RNA | 2.4ug | 9 | 1.2ug | Exome Cap- Transcriptome | Agilent v4 Oligos | SI_6702 | 3 |
| MO_1180  Tumor | 2+4+5 | 0.3+0.3+0.1  cm | 50-65% | DNA | 3.5ug | NA | 3ug | Exome | Agilent v4 Oligos | SI_6675 | 11 |
| MO_1180  Normal | Blood (Javed) | - | 0% | DNA | 26ug | NA | 3ug | Exome | Agilent v4 Oligos | SI_6676 | 12 |

**MO_1180 SNP Fingerprinting Analysis**

|  | SI_6691  (Poly(A)+Transcrip tome) | SI_6702 (Cap  Transcriptome) | SI_6675  (Tumor Exome) | SI_6676  (Normal Exome) |
| --- | --- | --- | --- | --- |
| SI_6691 (Poly(A)+  Transcriptome) | 162/162 (100%) | 159/159 (100%) | 158/158 (100%) | 157/157 (100%) |
| SI_6702  (Cap Transcriptome) | 159/159 (100%) | 160/160 (100%) | 158/158 (100%) | 158/158 (100%) |
| SI_6675  (Tumor Exome) | 158/158 (100%) | 158/158 (100%) | 162/162 (100%) | 160/160 (100%) |
| SI_6676  (Normal Exome) | 157/157 (100%) | 158/158 (100%) | 160/160 (100%) | 161/161 (100%) |

**MO**-**1180 Estimated Tumor Content = 70%**

Model with 2 Components

Model with 3 Components

-*en*

150

/

35 %

| / •  /  /  /  /  /  • ..../  /  /  .//  ......  . .,.,/ .  i/"'  -c..,•.,.  /  ,....,..  .•----- ------------  .- | |
| --- | --- |
|  |  |
| J._ | |

-*en*

150

.../

,./

/

/

,. 36 %

,./ •


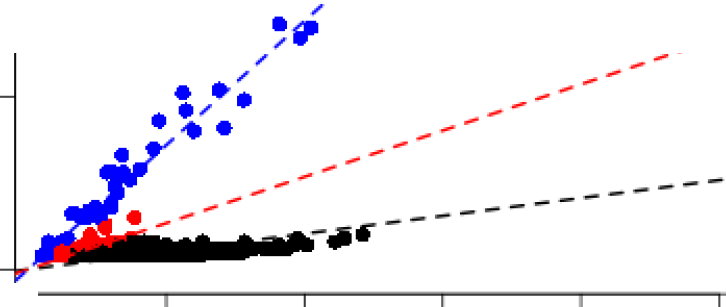


c

/

/

E

C) 100

- -

c

E

C) 100

/'·/

/./ /

f! f!

LL LL

50

/ / .

c c

·ma:: ·ma::

... 13 %

5 %

m m

> 50 >

6 %

?,

0 0

0 100 200 300 400 500 0

Total Fragments

100 200 300 400 500

Total Fragments

chr6q copy loss, including ***ARID1B*** and ***MYB***

**MO_1180 Copy Number Profile**


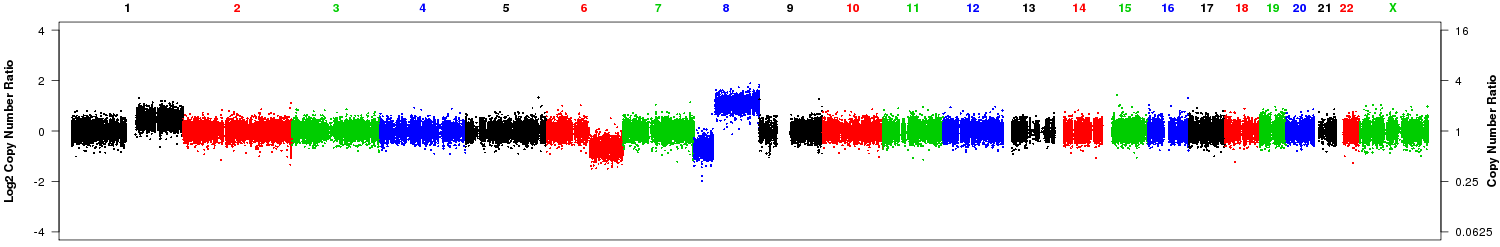


chr9 one copy loss of **CDKN2A** and ***CDKN2B***


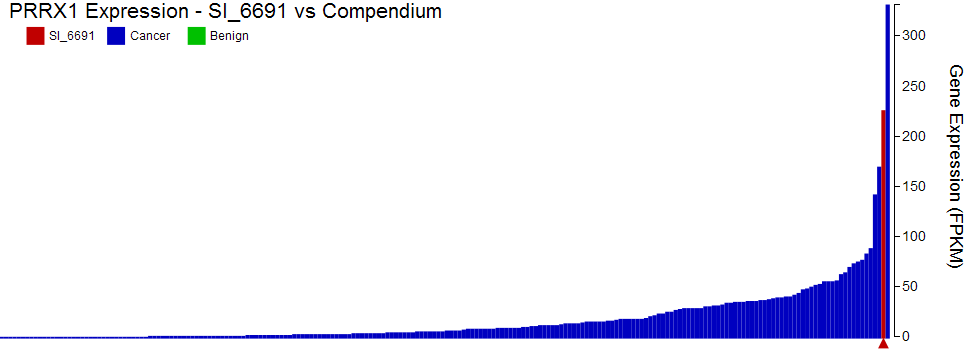

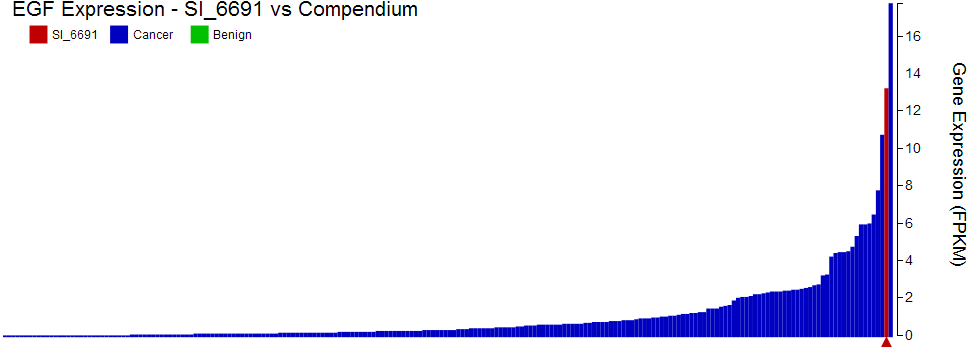


PRRX1: paired related homeobox 1, a transcription co-activator, enhancing the DNA-binding activity of serum response factor, a protein required for the induction of genes by growth and differentiation factors. The protein regulates muscle creatine kinase, indicating a role in the establishment of diverse mesodermal muscle types

**MO_1180 Expression Outliers**

**MO_1180 Copy Number Profile & LOH Plot**

Copy gain, shift in zygosity

Copy loss, LOH

Copy neutral LOH (UPD) No change


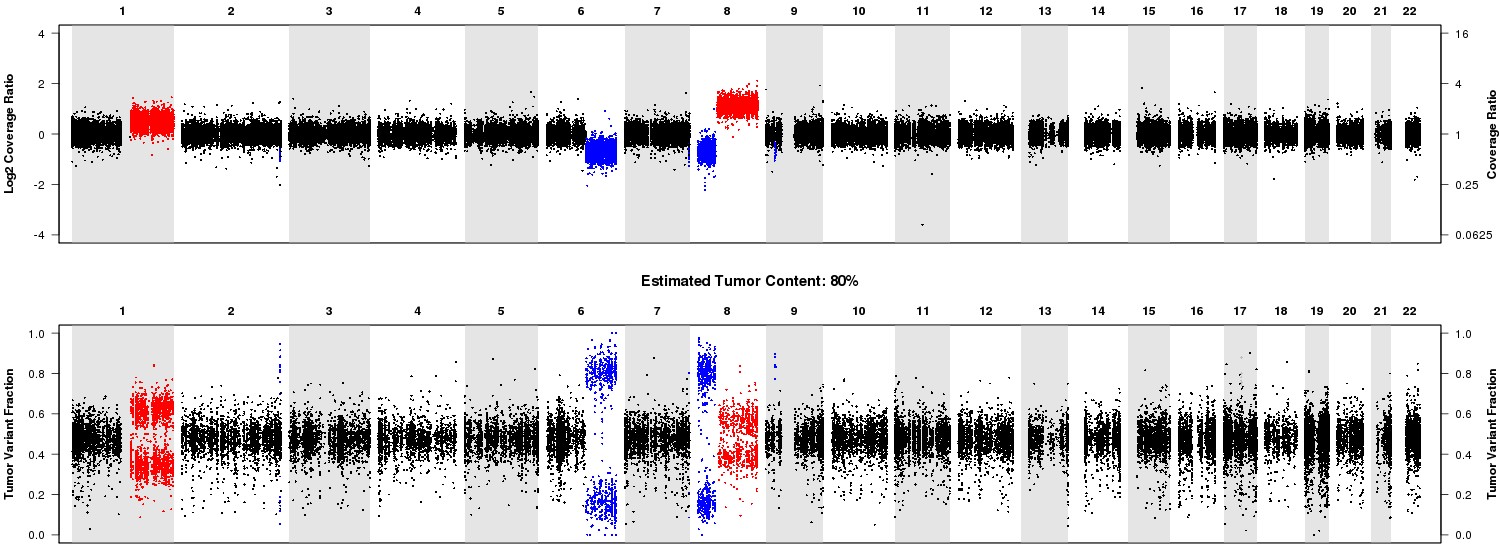


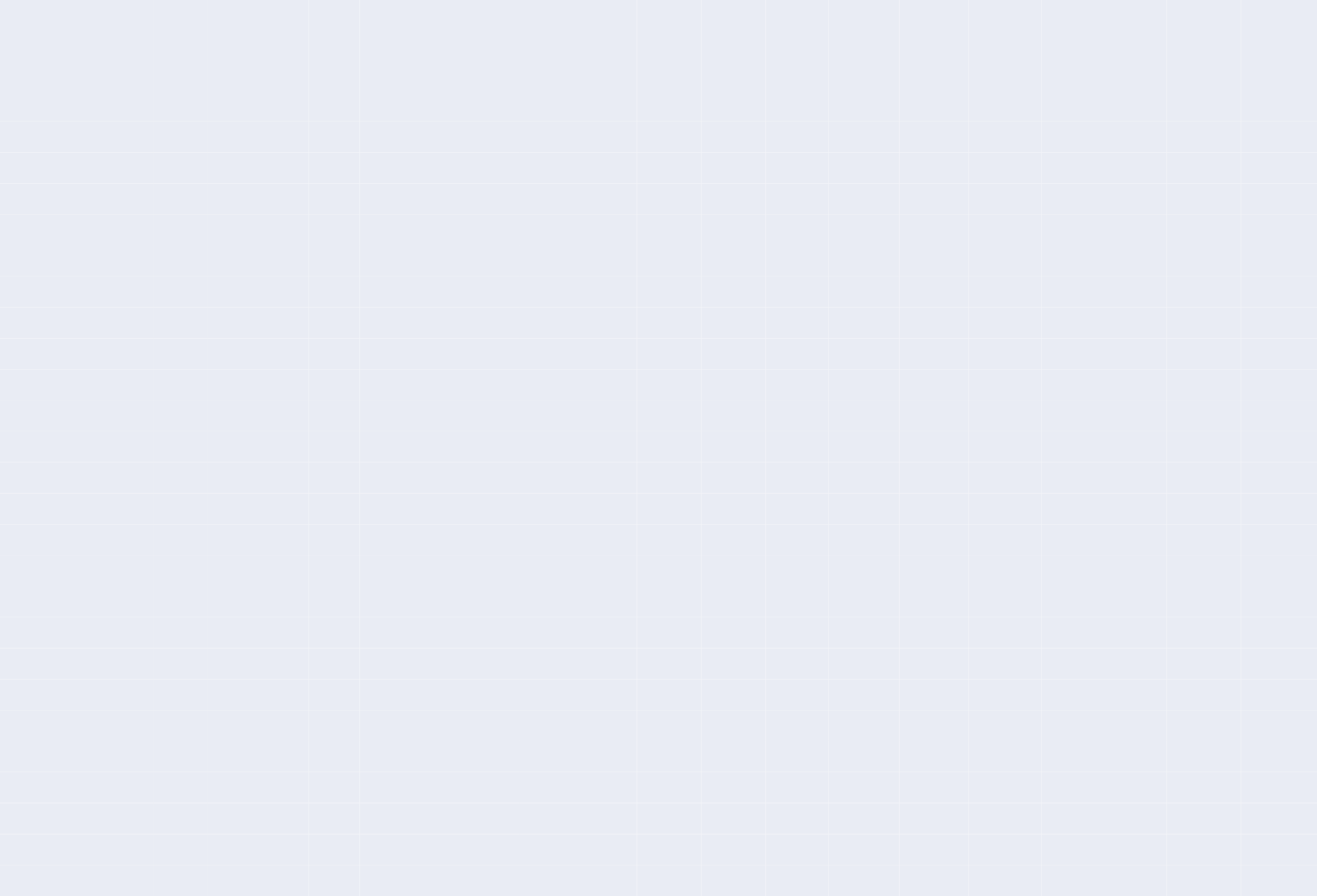


**MO_1180 = 26 Total somatic mutations**

| **Gene** | **Gene**  **Set** | **Amino Acid**  **Change** | **Chr** | **Coord** | **Ref** | **Var** | **Tumor Read Depth** | **Ref**  **Reads**  **> Q20**  **Tumor** | **Var**  **Reads**  **> Q20**  **Tumor** | **Var Allele Freq %** | **Normal Read Depth** | **Ref Reads > Q20**  **Normal** | **Gene Expr (FPKM)** | **Gene Expr percentile (Compendium)** | **COSMIC**  **@ Pos** | **COSMIC**  **+/- 6** |
| --- | --- | --- | --- | --- | --- | --- | --- | --- | --- | --- | --- | --- | --- | --- | --- | --- |
| TYK2 | Y | splicing | 19 | 10464717 | C | T | 184 | 111 | 71 | 39.0 | 210 | 209 | 39.2 | 18.1 | 0 | 0 |
| TMC6 | Y | p.R104C | 17 | 76121927 | G | A | 230 | 139 | 91 | 39.6 | 260 | 258 | 3.2 | 8.6 | 0 | 0 |
| CBLC | Y | splicing | 19 | 45295773 | T | A | 66 | 39 | 24 | 38.1 | 86 | 86 | 0.0 | 0.5 | 0 | 1 |
| EEF1A2 |  | p.P406R | 20 | 62120318 | G | C | 203 | 185 | 11 | 5.6 | 171 | 163 | 294.6 | 97.1 | 0 | 0 |
| SFPQ |  | p.A131P | 1 | 35658260 | C | G | 61 | 48 | 7 | 12.7 | 70 | 65 | 111.4 | 19.5 | 0 | 0 |
| WNK1 |  | p.R121G | 12 | 863092 | C | G | 336 | 305 | 17 | 5.3 | 365 | 348 | 49.6 | 87.1 | 0 | 0 |
| UBR5 |  | p.E1663X | 8 | 103298816 | C | A | 254 | 167 | 84 | 33.5 | 118 | 117 | 30.7 | 48.1 | 0 | 1 |
| MMS19 |  | p.A4V | 10 | 99258131 | G | A | 257 | 161 | 87 | 35.1 | 245 | 237 | 21.5 | 46.2 | 0 | 0 |
| GPNMB |  | p.T342I | 7 | 23306106 | C | T | 118 | 68 | 46 | 40.4 | 75 | 74 | 16.5 | 44.8 | 0 | 0 |
| TTC14 |  | p.Q635E | 3 | 180335296 | C | G | 10 | 4 | 6 | 60.0 | 10 | 10 | 10.0 | 40.0 | 0 | 0 |
| MED22 |  | p.S167X | 9 | 136208458 | G | T | 286 | 175 | 103 | 37.1 | 312 | 306 | 9.4 | 32.4 | 2 | 2 |
| ACACB |  | p.R1798C | 12 | 109684074 | C | T | 292 | 189 | 97 | 33.9 | 263 | 261 | 8.4 | 75.2 | 0 | 0 |
| TCP11L1 |  | p.A290T | 11 | 33083168 | G | A | 91 | 56 | 35 | 38.5 | 99 | 99 | 5.1 | 71.4 | 0 | 0 |
| KALRN |  | p.N369T | 3 | 124044846 | A | C | 399 | 340 | 18 | 5.0 | 390 | 357 | 4.5 | 46.2 | 0 | 0 |
| ZW10 |  | p.P255L | 11 | 113628545 | G | A | 71 | 41 | 28 | 40.6 | 58 | 57 | 3.4 | 40.0 | 0 | 0 |
| KIF21A |  | p.A891T | 12 | 39726726 | C | T | 359 | 233 | 121 | 34.2 | 293 | 291 | 2.1 | 24.3 | 0 | 0 |
| ALPK2 |  | p.A608P | 18 | 56246186 | C | G | 501 | 319 | 176 | 35.6 | 404 | 402 | 0.6 | 76.7 | 0 | 1 |
| SLC24A4 |  | p.V379A | 14 | 92922833 | T | C | 160 | 107 | 49 | 31.4 | 148 | 145 | 0.2 | 63.8 | 0 | 1 |
| AGBL1 |  | p.Q230H | 15 | 86800176 | C | T | 255 | 149 | 96 | 39.2 | 245 | 241 | 0.1 | 91.4 | 1 | 1 |
| COL6A6 |  | p.L105P | 3 | 130282161 | T | C | 310 | 282 | 15 | 5.1 | 227 | 214 | 0.1 | 63.3 | 0 | 0 |
| BMP15 |  | p.H83N | X | 50654030 | C | A | 100 | 29 | 69 | 70.4 | 107 | 101 | 0.0 | 0.5 | 0 | 0 |
| CYP4F12 |  | p.A376T | 19 | 15806756 | G | A | 198 | 130 | 67 | 34.0 | 198 | 196 | 0.0 | 0.5 | 0 | 0 |
| CRISP2 |  | p.H82Q | 6 | 49667542 | A | T | 114 | 61 | 51 | 45.5 | 94 | 93 | 0.0 | 0.5 | 0 | 0 |
| AC011467.1 |  | p.K80E | 19 | 22868206 | A | G | 12 | 4 | 8 | 66.7 | 5 | 5 | 0.0 | 0.5 | 1 | 2 |
| CTB-78H18.1.1 |  | p.S139P | 5 | 171201624 | T | C | 82 | 72 | 8 | 10.0 | 63 | 60 | 0.0 | 0.5 | 0 | 0 |
| CR1L |  | p.N402D | 1 | 207872595 | A | G | 37 | 31 | 6 | 16.2 | 9 | 9 | 0.0 | 0.5 | 0 | 2 |

**MO_1180 Indels**

| **Gene** | **ExonicFunc** | **Chr** | **Start** | **End** | **Ref** | **Obs** | **Var reads/ Total reads** | **Normal** | **Status** | **Comments** |
| --- | --- | --- | --- | --- | --- | --- | --- | --- | --- | --- |
| BCL9 | frameshift deletion | 1 | 147091501 | 147091501 | C | - | 5/591 | 1/364 | somatic? | PCR stutter/ homopolymer |

**MO_1180 Fusion candidates (PolyA+)**

**MO_1180 Fusion candidates (Capture)**

| **5' Gene** | **5' Chr** | **5' Coord** | **3' Gene** | **3' Chr** | **3' Coord** | **Spanning Reads** | **Spanning Mate Pairs** | **Spanning Mate Pairs w/ Fusion** |
| --- | --- | --- | --- | --- | --- | --- | --- | --- |
| NR4A3 | 9 | 102590322 | EWSR1 | 22 | 29692357 | 44 | 10 | 51 |

| **5' Gene** | **5' Chr** | **5' Coord** | **3' Gene** | **3' Chr** | **3' Coord** | **Spanning Reads** | **Spanning Mate Pairs** | **Spanning Mate Pairs w/ Fusion** |
| --- | --- | --- | --- | --- | --- | --- | --- | --- |
| NR4A3 | 9 | 102590322 | EWSR1 | 22 | 29692357 | 256 | 27 | 297 |
| NR4A3 | 9 | 102590321 | EWSR1 | 22 | 29692353 | 1 | 27 | 1 |
| SCMH1 | 1 | 41707647 | FOXO6 | 1 | 41847287 | 2 | 1 | 3 |
| MGC45800 | 4 | 183064065 | ODZ3 | 4 | 183245098 | 1 | 2 | 1 |
| FBXL8 | 16 | 67197129 | HSF4 | 16 | 67197356 | 1 | 14 | 0 |

ACTGGAACCTGGAGGGGAAGGGCTATATTGGGCTTGGACGCAGGGCATAT CATCAAACCATTCCACGGCAGCCTTGGCAGTGGGTGGGTCTTCATAGGAC

**MO_1180: EWSR1-NR4A3 Fusion**

**EWSR1**


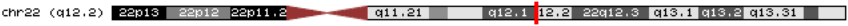


**NR4A3**


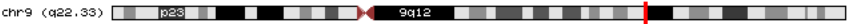

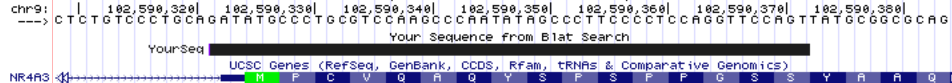

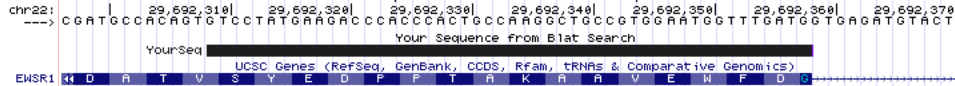


In-frame fusion

a.a.1

**MO_1180 Germline Variations**

| **Gene** | **Amino Acid**  **Change** | **Chr** | **Coord** | **Ref** | **Var** | **Tumor Read Depth** | **Ref**  **Reads**  **> Q20**  **Tumor** | **Var**  **Reads**  **> Q20**  **Tumor** | **Var Allele Freq %** | **Normal Allele Freq %** | **Normal Read Depth** | **Ref**  **Reads**  **> Q20**  **Normal** | **Var**  **Reads**  **> Q20**  **Normal** | **1000**  **Genome** |
| --- | --- | --- | --- | --- | --- | --- | --- | --- | --- | --- | --- | --- | --- | --- |
| FANCI | p.P55L | 15 | 89803950 | C | T | 129 | 63 | 66 | 51.2 | 59.5 | 79 | 32 | 47 | 0.03 |
| BLM | p.P868L | 15 | 91326099 | C | T | 163 | 90 | 72 | 44.4 | 47.1 | 87 | 46 | 41 | 0.05 |
| MSH6 | p.R158C | 2 | 48025764 | C | T | 129 | 66 | 62 | 48.4 | 48.0 | 75 | 39 | 36 | 0.05 |
| FANCE | p.S204L | 6 | 35423886 | C | T | 254 | 131 | 121 | 48.0 | 50.7 | 210 | 102 | 105 | 0.03 |
| FANCA | p.L123R | 16 | 89877395 | T | C | 148 | 77 | 69 | 47.3 | 47.4 | 135 | 70 | 63 |  |
| BARD1 | p.R658C | 2 | 215595164 | G | A | 218 | 126 | 89 | 41.4 | 45.4 | 195 | 106 | 88 | 0.01 |
| FANCG | p.S7F | 9 | 35079502 | G | A | 347 | 177 | 163 | 47.9 | 47.8 | 398 | 203 | 186 | 0.0014 |
| COL7A1 | p.R1202H | 3 | 48623625 | C | T | 414 | 200 | 204 | 50.5 | 46.7 | 377 | 195 | 171 | 0.0018 |
| GATA2 | p.W10C | 3 | 128205845 | C | A | 180 | 102 | 73 | 41.7 | 53.7 | 218 | 99 | 115 |  |
| MSH2 | p.V605L | 2 | 47702217 | G | C | 191 | 108 | 77 | 41.6 | 45.7 | 138 | 75 | 63 |  |
| FANCA | p.V6D | 16 | 89883007 | A | T | 300 | 147 | 143 | 49.3 | 42.6 | 298 | 166 | 123 | 0.05 |

**MO_1180: SEQUENCE ANALYSIS SUMMARY**

| **Event/Gene** | **Aberration** |
| --- | --- |
| chr1q, 8q | Copy gain |
| Chr6q, 8p | Copy loss |
| ***ARID1B, MYB, CDKN2A,***  ***CDKN2B*** | One copy loss |
| Human Pathogens | Not detected |
| Somatic mutations | Detected, unknown significance |
| ***EWSR1-NR4A3*** | Gene Fusion |

**MO_1222: PATIENT HISTORY**

Patient is a 46-year-old male with a diagnosis of extraskeletal myxoid chondrosarcoma,

The patient first presented with a left anterior thigh lesion in May 2001. He underwent a resection with positive margins, so he underwent a repeat resection on 5/25/01.

Immunohistochemical staining showed positivity for S100, P63, calponin, and EMA. Imaging performed in August 2001 demonstrated pulmonary nodules, but the patient decided to go on observation since they were all subcentimeter.

Imaging performed on 8/24/2008 demonstrated numerous noncalcified lung nodules. His pathology was reviewed again and was positive for EWS by split probe FISH. He started Ewing’s sarcoma treatment regimen containing Cytoxan, Adriamycin, vincristine of 1 cycle alternating with ifosfamide, etoposide on 8/24/08. Imaging performed on 10/21/2008 demonstrated disease progression after 2 total cycles. His treatment was changed to DTIC for 4 cycles, until imaging performed on 1/12/2009 demonstrated disease progression.

He initiated R1507 antibody to insulin-like growth factor receptor as part of clinical trial protocol on 3/5/09. The patient was taken off the study on 11/25/09 due to enlargement of 1 nodule. Since then, he has been on observation with periodic surveillance of scattered pulmonary nodules.

He underwent a lateral proximal thigh biopsy for MI-ONCOSEQ.

**MO_1222 Sequencing libraries**

| **Study ID** | **Tissue Block** | **Specimen size** | **Tumor content** | **Sample type** | **Yield** | **RNA RIN** | **Amount for Lib prep** | **Lib type** | **Exome platform** | **Library ID** | **Barcode** |
| --- | --- | --- | --- | --- | --- | --- | --- | --- | --- | --- | --- |
| MO_1222  Tumor | 1+2 | 0.2+0.1cm | 40-50% | RNA | 2.8ug | 9.4 | 1.4ug | Poly(A)+ Transcriptome | - | SI_7009 | 1 |
| MO_1222  Tumor | 1+2 | 0.2+0.1cm | 40-50% | RNA | 2.8ug | 9.4 | 1.4ug | Exome Cap-  Transcriptome | Agilent v4  Oligos | SI_7124 | 4 |
| MO_1222  Tumor | 1+2 | 0.2+0.1cm | 40-50% | DNA | 3.9ug | NA | 3ug | Exome | Agilent v4 Oligos | SI_7099 | 1 |
| MO_1222  Normal | Blood  (Javed) | - | 0% | DNA | 34ug | NA | 3ug | Exome | Agilent v4  Oligos | SI_7100 | 2 |

**MO_1222 Copy Number Profile**

No significant copy gain or loss detected


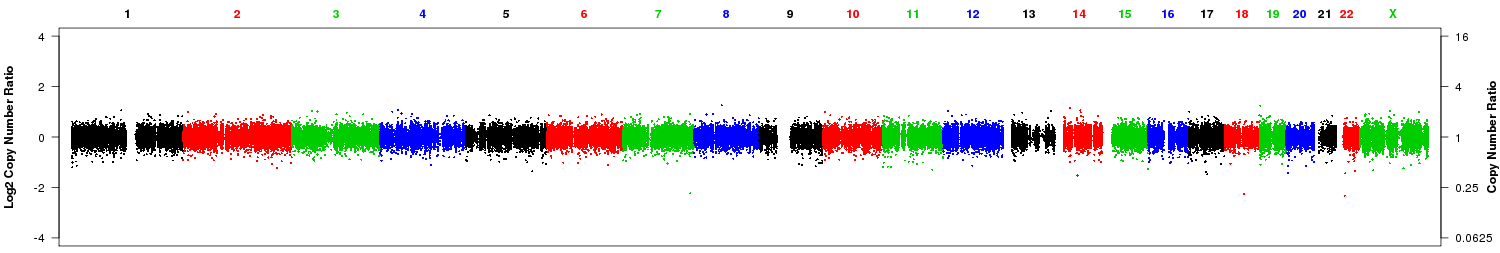


**MO_1222 Copy Number Profile & LOH Plot**

Copy gain, shift in zygosity

Copy loss, LOH

Copy neutral LOH (UPD) No change


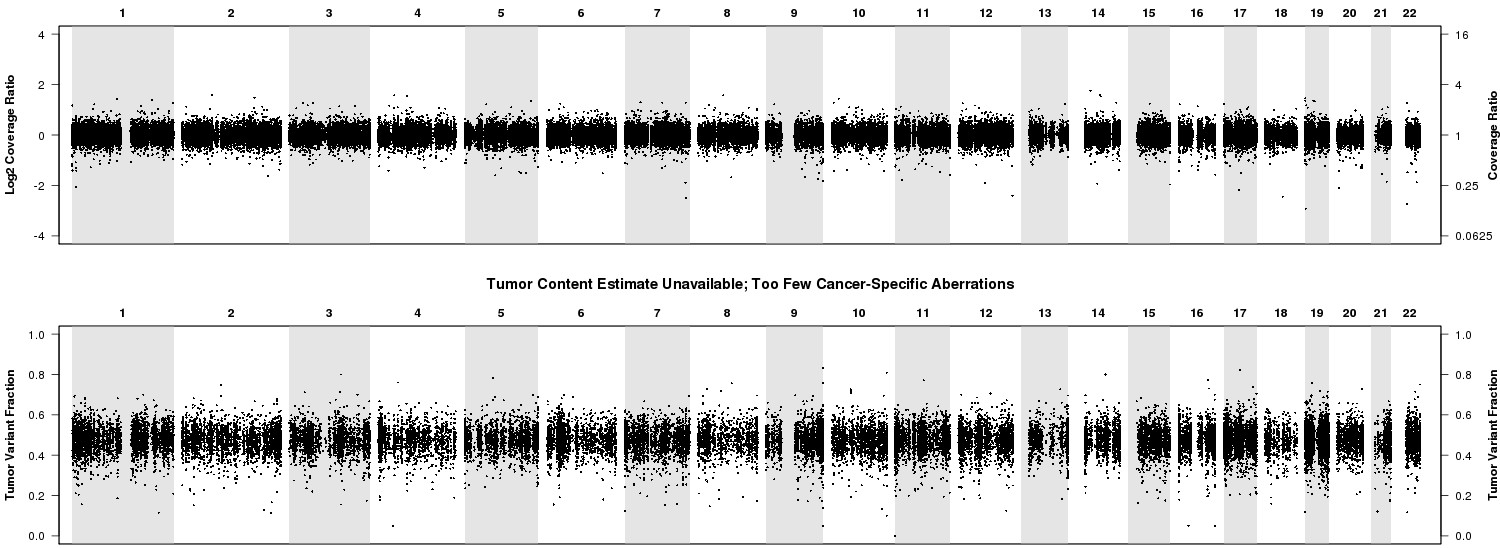


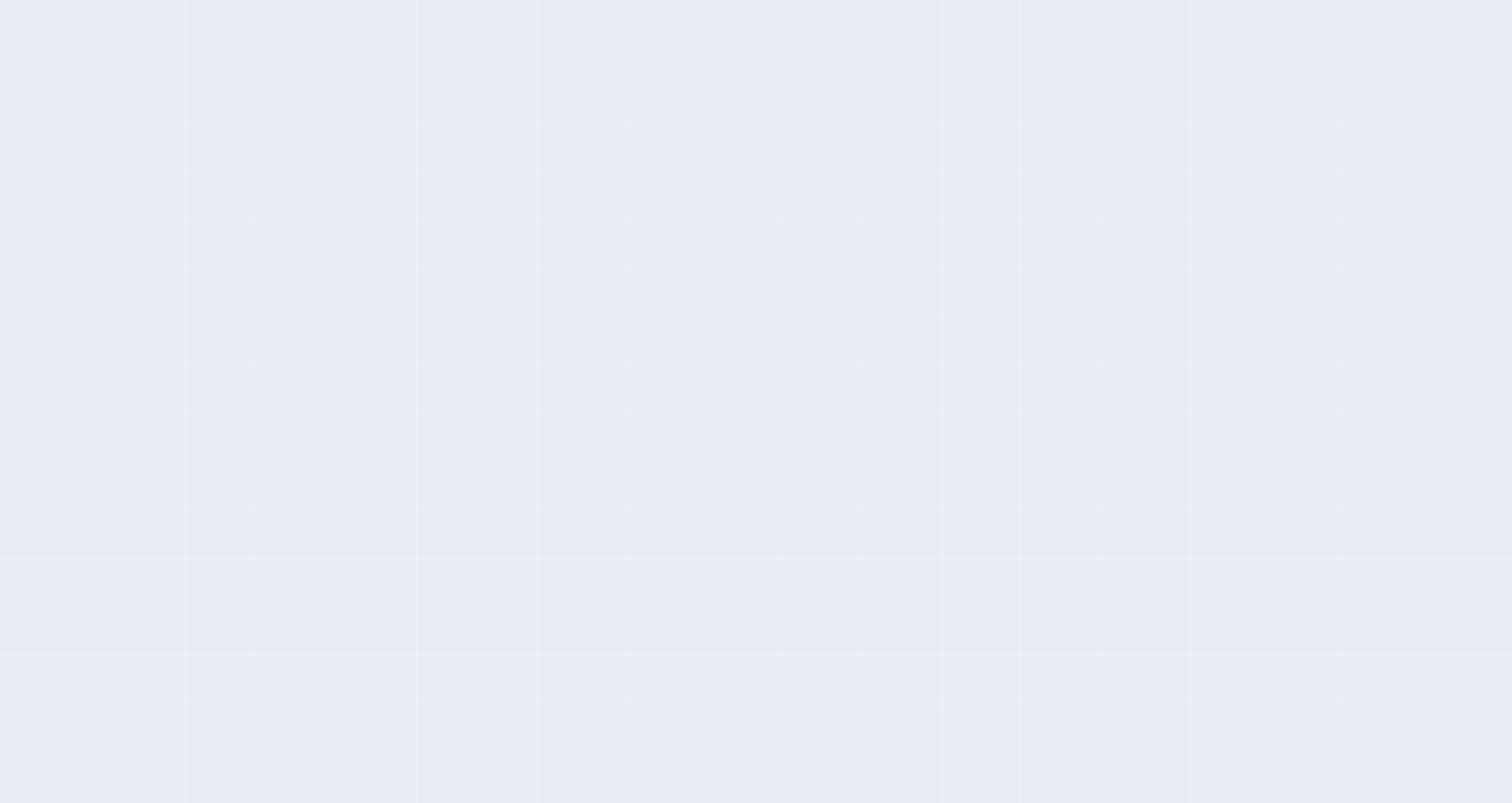


**MO_1222 = 15 Total somatic mutations**

| **Gene** | **Gene Set** | **Amino Acid Change** | **Chr** | **Coord** | **Ref** | **Var** | **Tumor Read Depth** | **Ref**  **Reads**  **> Q20**  **Tumor** | **Var**  **Reads**  **> Q20**  **Tumor** | **Var Allele Freq %** | **Normal Read Depth** | **Ref Reads > Q20**  **Normal** | **Gene Expr (FPKM)** | **Gene Expr percentile Compendium** | **COSMIC**  **@ Pos** | **COSMIC**  **+/- 6** |
| --- | --- | --- | --- | --- | --- | --- | --- | --- | --- | --- | --- | --- | --- | --- | --- | --- |
| MYH9 |  | p.V1930M | 22 | 36678809 | C | T | 290 | 250 | 20 | 7.4 | 219 | 214 | 177.6 | 63.8 | 0 | 0 |
| ATIC |  | splicing | 2 | 216213455 | G | A | 16 | 10 | 6 | 37.5 | 5 | 5 | 29.2 | 5.0 | 0 | 0 |
| CRY1 |  | p.P504S | 12 | 107391147 | G | A | 133 | 70 | 59 | 45.7 | 97 | 96 | 25.7 | 98.9 | 0 | 0 |
| VARS |  | p.R1119H | 6 | 31747246 | C | T | 305 | 256 | 30 | 10.5 | 195 | 185 | 14.9 | 14.0 | 0 | 0 |
| DACT3 |  | p.K624X | 19 | 47151759 | T | A | 532 | 489 | 29 | 5.6 | 396 | 389 | 12.8 | 93.9 | 0 | 0 |
| KIAA2026 |  | p.I1401T | 9 | 5921794 | A | G | 287 | 169 | 110 | 39.4 | 162 | 160 | 10.3 | 85.7 | 0 | 4 |
| TAP2 |  | p.I288K | 6 | 32803013 | A | T | 186 | 114 | 66 | 36.7 | 141 | 135 | 7.6 | 30.8 | 0 | 2 |
| ZNF142 |  | p.S511Y | 2 | 219509707 | G | T | 292 | 188 | 88 | 31.9 | 205 | 199 | 5.8 | 39.1 | 0 | 2 |
| DAAM1 |  | p.Y833H | 14 | 59821993 | T | C | 162 | 105 | 51 | 32.7 | 100 | 99 | 3.5 | 8.6 | 0 | 2 |
| CCDC165 |  | p.P1476T | 18 | 8824977 | C | A | 800 | 467 | 297 | 38.9 | 627 | 611 | 2.3 | 54.5 | 0 | 0 |
| MUC3A |  | p.E702A | 7 | 100608370 | A | C | 401 | 359 | 28 | 7.2 | 274 | 262 | 0.3 | 31.5 | 0 | 0 |
| UGT8 |  | p.A18V | 4 | 115544089 | C | T | 226 | 132 | 80 | 37.7 | 123 | 121 | 0.1 | 12.5 | 0 | 1 |
| ADH7 |  | p.F379S | 4 | 100336659 | A | G | 100 | 58 | 41 | 41.4 | 86 | 85 | 0.0 | 0.4 | 0 | 0 |
| ENSG00000250264 |  | p.I288K | 6 | 32803013 | A | T | 186 | 114 | 66 | 36.7 | 141 | 135 |  |  | 0 | 2 |

**MO_1222 Fusion candidates (PolyA+)**

| **5' Gene** | **5' Chr** | **5' Coord** | **3' Gene** | **3' Chr** | **3' Coord** | **Gene Set** | **Spanning Reads** | **Spanning Mate Pairs** | **Spanning Mate Pairs w/ Fusion** |
| --- | --- | --- | --- | --- | --- | --- | --- | --- | --- |
| NR4A3 | 9 | 102584688 | EWSR1 | 22 | 29693816 | Y | 83 | 27 | 89 |
| NR4A3 | 9 | 102584688 | EWSR1 | 22 | 29694722 | Y | 9 | 27 | 13 |
| NR4A3 | 9 | 102585630 | EWSR1 | 22 | 29693816 | Y | 1 | 27 | 1 |
| NR4A3 | 9 | 102586687 | EWSR1 | 22 | 29693093 | Y | 17 | 62 | 16 |
| NR4A3 | 9 | 102587940 | EWSR1 | 22 | 29692357 | Y | 75 | 62 | 94 |

Note: EWSR1-NR4A3 fusions are reciprocal

**MO_1222 Fusion candidates (Capture)**

| **5' Gene** | **5' Chr** | **5' Coord** | **3' Gene** | **3' Chr** | **3' Coord** | **Gene Set** | **Spanning Reads** | **Spanning Mate Pairs** | **Spanning Mate Pairs w/ Fusion** |
| --- | --- | --- | --- | --- | --- | --- | --- | --- | --- |
| **NR4A3** | 9 | 102587940 | **EWSR1** | 22 | 29692357 | Y | 48 | 43 | 83 |
| TANC2 | 17 | 61278316 | RP5-968J1.1.1 | 20 | 1791320 |  | 3 | 3 | 1 |

TCCAGGCTCAGGGCCCGGGCTTGCCGGGCTGCCCTCTTCCGCAGTGGGCT CATCAAACCATTCCACGGCAGCCTTGGCAGTGGGTGGGTCTTCATAGGAC

**MO_1222: EWSR1-NR4A3 Fusion**

EWSR1 exon11(29692227-29692357); NR4A3 exon2(102587939-102588113)

**EWSR1**


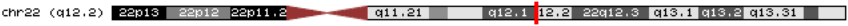


**NR4A3**


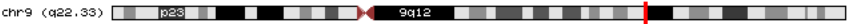

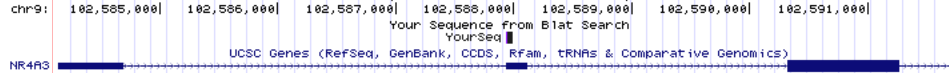

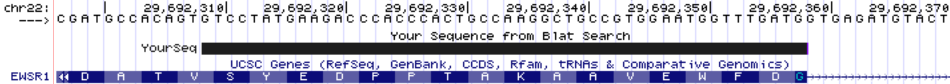


EWSR1 in-frame fused to NR4A3 5’ UTR

M N K R T G Q P M I H I Y L D K E T G K P K G D A T V S Y E D P P T A K A A V E W F D E P T A E E G S P A S P G P E P G P L A V P G S T A G A S P R R T S A P P T L S A S A G E T P S P T I Q R A R Y P P G P H H L F S S Q D F I P Y **M** H D S I R F G N V D M P C V Q A Q Y S P S P P G S S Y A A Q T Y S S E Y T T E I M N P


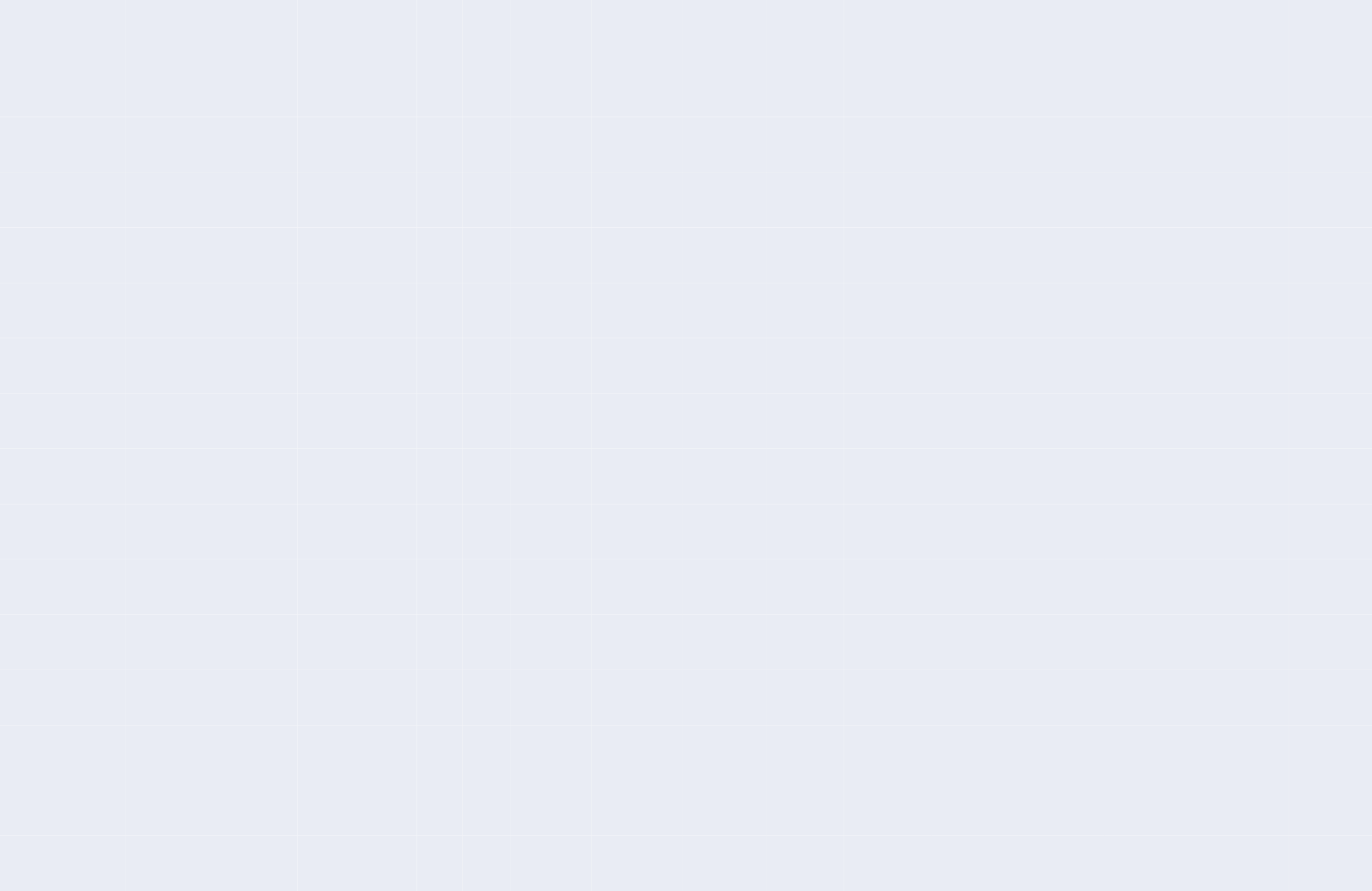


**MO_1222 Germline Variations**

| **Gene** | **Amino Acid Change** | **Chr** | **Coord** | **Ref** | **Var** | **Tumor Read Depth** | **Ref Reads > Q20**  **Tumor** | **Var Reads**  **> Q20**  **Tumor** | **Var Allele Freq %** | **Normal Allele Freq %** | **Normal Read Depth** | **Ref Reads**  **> Q20**  **Normal** | **Var Reads**  **> Q20**  **Normal** | **1000**  **Genome** | **LOH** |
| --- | --- | --- | --- | --- | --- | --- | --- | --- | --- | --- | --- | --- | --- | --- | --- |
| MSH2 | p.G322D | 2 | 47643457 | G | A | 74 | 44 | 27 | 38.0 | 47.2 | 54 | 28 | 25 | 0.01 | No |
| TSC1 | p.L116V | 9 | 135800991 | A | C | 75 | 34 | 38 | 52.8 | 43.9 | 68 | 37 | 29 | 0.0005 | No |
| KIT | p.N400S | 4 | 55575673 | A | G | 140 | 63 | 68 | 51.9 | 45.8 | 75 | 39 | 33 | 0.0023 | No |
| BLM | p.S1380R | 15 | 91358395 | T | G | 303 | 158 | 136 | 46.3 | 45.7 | 169 | 88 | 74 |  | No |
| MSH6 | p.V878A | 2 | 48027755 | T | C | 167 | 79 | 82 | 50.9 | 56.8 | 131 | 54 | 71 | 0.01 | No |
| PARK2 | p.R275W | 6 | 162206852 | G | A | 194 | 83 | 106 | 56.1 | 42.6 | 128 | 70 | 52 | 0.0005 | No |
| PARK2 | p.P437L | 6 | 161771219 | G | A | 196 | 92 | 92 | 50.0 | 39.7 | 127 | 73 | 48 | 0.02 | No |
| ALK | p.V476A | 2 | 29543736 | A | G | 123 | 59 | 59 | 50.0 | 45.8 | 76 | 39 | 33 | 0.02 | No |
| BRCA2 | p.I62V | 13 | 32949533 | A | G | 24 | 11 | 12 | 52.2 | 55.6 | 19 | 8 | 10 | 0.0046 | No |
| PMS2 | p.T597S | 7 | 6026607 | T | A | 221 | 106 | 99 | 48.3 | 47.2 | 153 | 76 | 68 | 0.0046 | No |
| GALNT12 | p.E119V | 9 | 101570336 | A | T | 114 | 54 | 53 | 49.5 | 45.4 | 92 | 47 | 39 | 0.04 | No |
| TERT | p.A279T | 5 | 1294166 | C | T | 552 | 265 | 249 | 48.4 | 50.1 | 443 | 206 | 207 | 0.01 | No |
| TSC2 | p.W477C | 16 | 2114407 | C | T | 207 | 94 | 104 | 52.5 | 46.6 | 145 | 71 | 62 | 0.04 | No |
| FANCM | p.T1600I | 14 | 45658024 | C | T | 21 | 12 | 9 | 42.9 | 42.9 | 18 | 8 | 6 | 0.01 | No |

**MO_1222: SEQUENCE ANALYSIS SUMMARY**

| **Event/Gene** | **Aberration** |
| --- | --- |
| Human Pathogens | Not detected |
| Gene copy number | No significant changes |
| Point mutations | Detected, unknown significance |
| ***EWSR1-NR4A3*** | Gene fusion |

Patient is a 55 y.o. male with metastatic extraskeletal myxoid chondrosarcoma.

**MO_1381: PATIENT HISTORY**

In 1992, patient noticed pain and discomfort in the left thigh accompanied by a mass. In February and March of 1993, biopsies revealed EMC. He underwent resection. In fall of 1999, he had a local recurrence and underwent radical resection of the mass in the left medial thigh in September 1999. Pathology was consistent with EMC. Following surgery, he was placed on observation.

Chest CT in March of 2008 revealed multiple bilateral pulmonary nodules. In May 2008, patient underwent a right upper lobe thoracoscopic wedge biopsy of the lung nodule with pathology revealing metastatic extraskeletal myxoid chondrosarcoma.

In September 2009, chest CT revealed progression of nodule as well as increased size in the left thigh mass. In November 2009, patient initiated Cytoxan and rapamycin as part of clinical study. From November 2011 through January 2012, he underwent radiation to the left thigh mass. Cytoxan was on hold during this time. He continued on rapamycin. Imaging in June of 2014 indicated progression in size of lung nodules, so rapamycin was stopped.

Patient underwent a lung biopsy for MIONCOSEQ.

# Libraries

**MO_1381: SEQUENCING SAMPLES**

| **Study ID** | **Tissue Block** | **Specimen size** | **Tumor content** | **Sample type** | **Yield** | **RNA RIN** | **Amount for Lib prep** | **Lib type** |
| --- | --- | --- | --- | --- | --- | --- | --- | --- |
| MO_1381  Tumor | 2+5+6+7 | 0.1+0.2+  0.2+0.1cm | 20-40% | RNA | 1.1ug | 7.2 | 0.9ug | Exome Cap- Transcriptome |
| MO_1381  Tumor | 2+5+6+7 | 0.1+0.2+  0.2+0.1cm | 20-40% | DNA | 2.7ug | NA | 2.5ug | Exome |
| MO_1381  Normal | Blood | - | 0% | DNA | 32ug | NA | 3ug | Exome |

Limited RNA, only tumor and normal exome and capture RNA libraries were analyzed.

**M0_1381: SEQUENCING SAMPLES- QC**


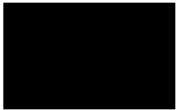
Sample Quality

*.*


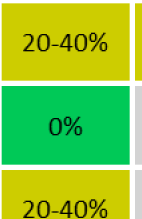

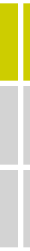


*••:*

24 %

•*:*

<30%

RIN

(SNP Fingerprinting)

Sequencing Quality Library Quality

Sample Identity

Sl_8679

Library

Sl_8678

(Tumor Exome)

| Number of Pass Filter Clusters | | | | Alignment Percentage | Mean Coverage | Percent On Target | PCR  Duplication Percentage | Covered SNPs | Mirnmum Concordance with other libraries | | Maximum Concordance with other patients | |
| --- | --- | --- | --- | --- | --- | --- | --- | --- | --- | --- | --- | --- |
|  | | | |  |  |  |  |  |  | | - | - |
|  | | | |  |  |  |  |  | - - - | - | - - -- | |
| - - | - - - | - | - | - -- - - |  |  | | - - - | - - - | - | - - -- | |

(Normal Exome)

Sl _ 8697 (Capture RNA)

Modelwith 2 Component s

1 -----------

r --M-od-elw-ith,-,3-,C,-,o-,m,-,p-,o,-n,-e,n'-,t,-s,-,-,'-,/

120

**26 %**

100

c

Ill

LL

**12 %**

c 60

-

**40**

c 60 -- 11 %

**.!S!** ,,,,•" .....

. .

. -

**40** ,' • ................

5 %

,, ......

***.....r.•***

**20** ,;........ : ---

**-ti••** -

,,'.. • ..... ***...tl-;t"***

20 **(&fH- - - ---**

- **5 %**

o !!'I

**t iil:';0;',**---

o *2*

L,.- --r -.-- --r----r--r-'

0 100

200 500 0 100 200 JOO 500

**Total Fragments Total Fragments**

Color Legend:

-

I I

I I

QC Pass MarginalPass QC Fail

Pass After Review

# Copy Number Profile

**MO_1381: SEQUENCE ANALYSIS SUMMARY**


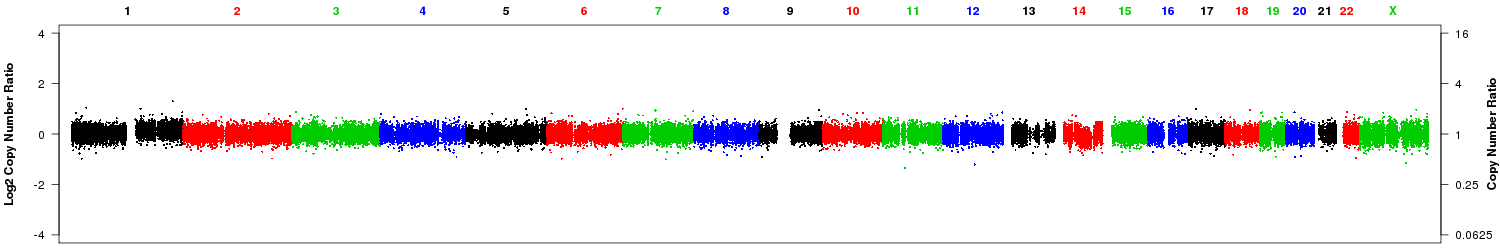


No focal amplification or deletion detected

# Copy Number Profile & LOH Plot

**MO_1381: SEQUENCE ANALYSIS SUMMARY**

Copy gain, shift in zygosity Copy loss, LOH

Copy neutral LOH (UPD)

No change


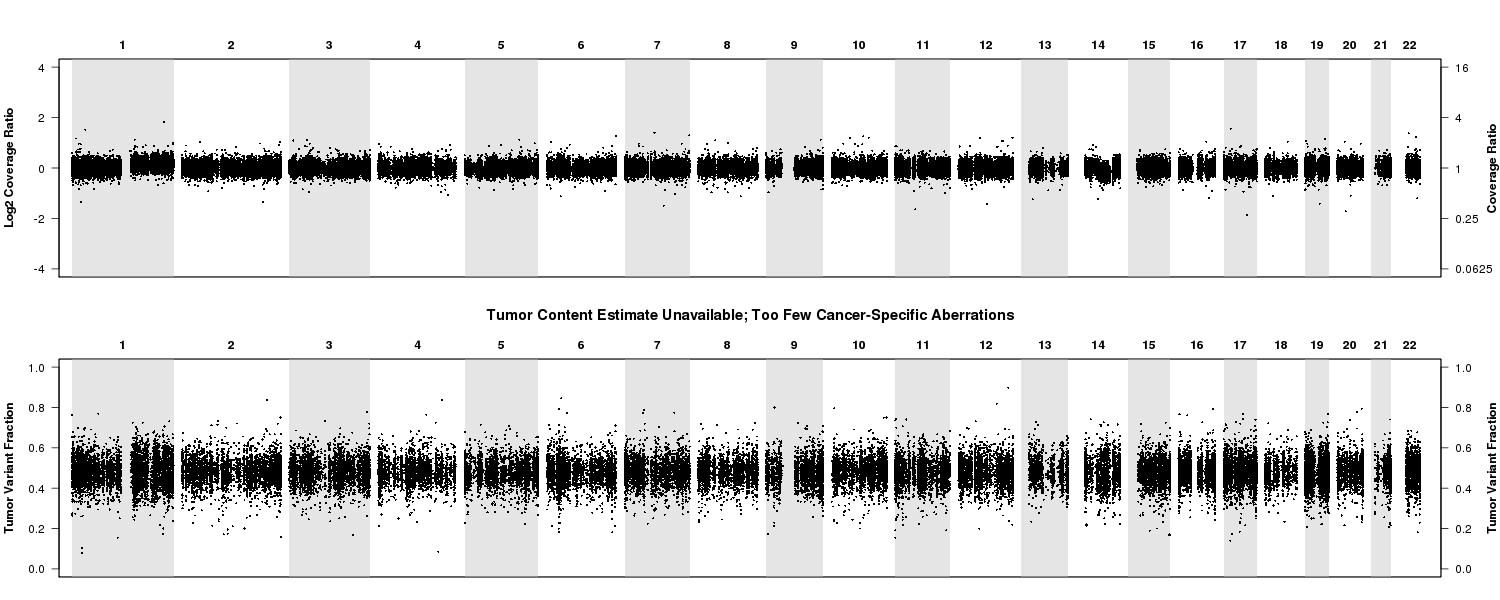


# Total somatic mutations: 8

**MO_1381: SEQUENCE ANALYSIS SUMMARY**

| **Gene** | **Gene Set** | **Amino Acid Change** | **Chr** | **Coord** | **Ref** | **Var** | **Tumor Read Depth** | **Ref Reads**  **> Q20**  **Tumor** | **Var Reads**  **> Q20**  **Tumor** | **Var Allele Freq %** | **Normal Read Depth** | **Ref Reads**  **> Q20**  **Normal** | **Gene Expr (FPKM)** | **Gene Expr percentile Compendium** | **COSMIC**  **@ Pos** | **COSMIC**  **+/- 6** |
| --- | --- | --- | --- | --- | --- | --- | --- | --- | --- | --- | --- | --- | --- | --- | --- | --- |
| BTK | Y | p.E280D | X | 100614335 | C | A | 106 | 89 | 9 | 9.2 | 101 | 96 | 3.0 | 52.6 | 0 | 0 |
| ZNF804A |  | p.Y446X | 2 | 185801461 | T | G | 286 | 249 | 27 | 9.8 | 249 | 245 | 0.3 | 54.9 | 0 | 2 |
| NPHP3 |  | p.E492K | 3 | 132423092 | C | T | 241 | 212 | 21 | 9.0 | 211 | 206 | 14.7 | 63.1 | 0 | 1 |
| KIF24 |  | p.G331V | 9 | 34290307 | C | A | 142 | 122 | 15 | 11.0 | 154 | 149 | 3.6 | 55.5 | 1 | 1 |
| C9orf47 |  | p.A34P | 9 | 91606010 | G | C | 440 | 379 | 47 | 11.0 | 377 | 366 | 0.8 | 70.1 | 0 | 0 |
| OR4D10 |  | p.T241S | 11 | 59245624 | C | G | 170 | 145 | 13 | 8.2 | 138 | 135 | 0.0 | 65.7 | 0 | 0 |
| GAL3ST3 |  | p.S28T | 11 | 65812804 | C | G | 151 | 132 | 17 | 11.4 | 140 | 136 | 1.0 | 85.9 | 0 | 0 |
| CCDC102B |  | p.Q192X | 18 | 66504574 | C | T | 274 | 227 | 28 | 11.0 | 242 | 230 | 18.6 | 92.4 | 0 | 0 |

***Fusion Candidates (Capture)***

**MO_1381: SEQUENCE ANALYSIS SUMMARY**

| **5' Gene** | **5' Chr** | **5' Coord** | **5 ' FPKM** | **3' Gene** | **3' Chr** | **3' Coord** | **3 ' FPKM** | **Gene Set** | **Spanning Reads** | **Spanning Mate Pairs** | **Spanning Mate Pairs w/ Fusion** |
| --- | --- | --- | --- | --- | --- | --- | --- | --- | --- | --- | --- |
| NR4A3 | 9 | 102590322 | 7.9 | EWSR1 | 22 | 29692357 | 56.7 | Y | 179 | 256 | 246 |
| CCDC11 | 18 | 47753746 | 19.4 | MYO5B | 18 | 47581747 | 15.0 |  | 5 | 6 | 5 |
| CCDC11 | 18 | 47753767 | 19.4 | MYO5B | 18 | 47581747 | 15.0 |  | 17 | 6 | 15 |
| TMEM135 | 11 | 86947725 | 31.2 | DLG2 | 11 | 84865694 | 7.4 |  | 29 | 1 | 5 |
| SAMD5 | 6 | 147830522 | 3.5 | SASH1 | 6 | 148711269 | 48.0 |  | 5 | 2 | 4 |
| C5orf56 | 5 | 131755631 | 11.5 | RAD50 | 5 | 131894975 | 24.3 | Y | 1 | 1 | 2 |

## MO_1381: EWSR1-NR4A3 Fusion

**MO_1381: SEQUENCE ANALYSIS SUMMARY**

ACTGGAACCTGGAGGGGAAGGGCTATATTGGGCTTGGACGCAGGGCATAT CATCAAACCATTCCACGGCAGCCTTGGCAGTGGGTGGGTCTTCATAGGAC

NR4A3 exon3 (102590321-102591274) EWSR1 exon11(29692227-29692357)

**EWSR1**


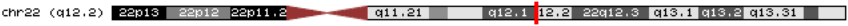


**NR4A3**


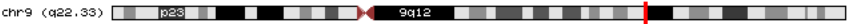

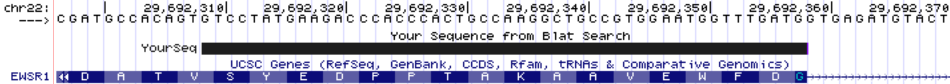

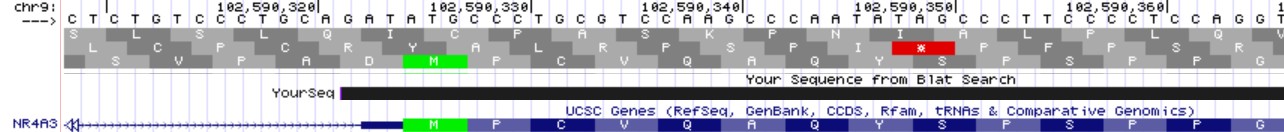


EWSR1 in-frame fused to NR4A3

# Germline Variations

**MO_1381: SEQUENCE ANALYSIS SUMMARY**

| **Gene** | **Amino Acid Change** | **Chr** | **Coord** | **Ref** | **Var** | **Tumor Read Depth** | **Ref Reads**  **> Q20**  **Tumor** | **Var Reads**  **> Q20**  **Tumor** | **Var Allele Freq**  **%** | **Normal Read Depth** | **Ref Reads**  **> Q20**  **Normal** | **Var Reads**  **> Q20**  **Normal** | **Normal Allele Freq %** | **1000**  **Genome** | **LOH** |
| --- | --- | --- | --- | --- | --- | --- | --- | --- | --- | --- | --- | --- | --- | --- | --- |
| RAD54L | p.D21G | 1 | 46714242 | A | G | 343 | 180 | 153 | 46.0 | 285 | 145 | 132 | 47.7 | 0.0037 | No |
| CYP21A2 | UNKNOWN | 6 | 31974671 | G | A | 243 | 152 | 84 | 35.6 | 205 | 133 | 67 | 33.5 |  | No |
| MLH3 | p.E624Q | 14 | 75514489 | C | G | 355 | 204 | 145 | 41.6 | 299 | 160 | 133 | 45.4 | 0.01 | No |
| FANCI | p.P55L | 15 | 89803950 | C | T | 225 | 113 | 104 | 47.9 | 192 | 91 | 97 | 51.6 | 0.03 | No |
| FANCI | p.A1311S | 15 | 89859634 | G | T | 345 | 175 | 139 | 44.3 | 300 | 156 | 112 | 41.8 |  | No |
| FANCA | p.M717I | 16 | 89838086 | C | A | 163 | 93 | 59 | 38.8 | 139 | 75 | 56 | 42.8 | 0.03 | No |
| LAMA3 | p.P1208T | 18 | 21424991 | C | A | 218 | 101 | 104 | 50.7 | 173 | 91 | 68 | 42.8 | 0.04 | No |

**MO_1381: SEQUENCE ANALYSIS SUMMARY**

| **Mutation class** | **Gene/Aberration** | **Potential Therapies/*Clinical Trials (*Contingent on meeting study eligibility criteria )** |
| --- | --- | --- |
| Copy number variation | No focal amplification or deletion detected |  |
| Somatic point mutations (8 Total) | Detected, unknown significance |  |
| Insertions/deletions (indels) | N/A |  |
| Gene fusions | EWSR1-NR4A3  (NR4A3 is also known as CHN) |  |
| Outlier expression | N/A |  |
| Germline variants | N/A |  |
| Pathogens | N/A |  |

**PRECISION TUMOR BOARD DISCUSSION/INTERPRETATION**

- *Aberrations that may relate to standard of care:* N/A
- *Aberrations that may make patient eligible for an open clinical trial or other therapies:*

Patient’s tumor harbors the EWSR1-NR4A3 gene fusion. A recent report confirms the therapeutic activity of sunitinib in extraskeletal myxoid chondrosarcoma (EMC). Genotype/phenotype analyses supported a correlation between response and EWSR1-NR4A3 fusion (*Eur J Cancer*. 2014 Jun;50(9):1657-64). Patient’s tumor expresses several targets of sunutinib (KDR, PDGFRA/B, KIT, RET, FLT1, FLT4); sunutinib could be considered here.

- *Germline mutations/family history- implications for disclosure:*

Patient MO-1381 has a personal history of sarcoma and family history of early onset breast cancer, raising the possibility of mutation in the TP53 gene. Research analysis of TP53 did not identify a germline mutation; however this research analysis may miss some mutations. Genetic counseling and clinical testing can be considered as the identification of a mutation may have implications for cancer surveillance for this patient and their blood relatives. Please contact our Clinical Cancer Genetics Service at 734-763-2532; [CancerGenetics@med.umich.edu](mailto:CancerGenetics@med.umich.edu) or by pager at 734-763-7672 #90132 with questions or to arrange a Clinical Cancer Genetics Consult.

- *Other informative results:*

EWSR1-NR4A3 gene fusion is found in about 75% of extraskeletal myxoid chondrosarcomas.

**MO_1582: PATIENT HISTORY**

## 37 year old man with metastatic extraskeletal myxoid chondrosarcoma involving the lungs

- 06/2014: Chest imaging showed multiple pulmonary nodules concerning for metastatic disease. Chest CT confirmed the presence of multiple bilateral lung nodules measuring up to 1.7 cm.
- 7/14/14: Biopsy showed extraskeletal myxoid chondrosarcoma.
- 10/2014: Started pazopanib for progressive disease.
- 12/2015: D/C pazopanib for disease progression.
- 1/2016-2/2016: Doxorubicin 75 mg/sq m x 2 cycles.
- 03/03/2016: Progression of EMC on doxorubicin. Referred for Mi-Oncoseq.

Patient underwent Pleural Nodal Biopsy for MI-ONCOSEQ Profiling Study

**MO_1582: SEQUENCING LIBRARIES**

| **Library ID** | **Block/Core** | **Tumor** | **Preservation** | **Biopsy Site** | **Isolate ID** | **Molecule** | **RIN** | **Capture Type** | **Library Type** | **QC Status** |
| --- | --- | --- | --- | --- | --- | --- | --- | --- | --- | --- |
| **SI_13112** | 1 | N | Refrigeration |  | 6950 | DNA |  | Onco1500 v2 | Capture  Genome | PASS |
| **SI_13082** | 3, 4, 6 | T | Frozen | Lung | 6884 | RNA | 8.2 | Exome | Capture Transcriptome | PASS |
| **SI_13111** | 3, 4, 6 | T | Frozen | Lung | 6941 | DNA |  | Onco1500 v2 | Capture Genome | PASS |


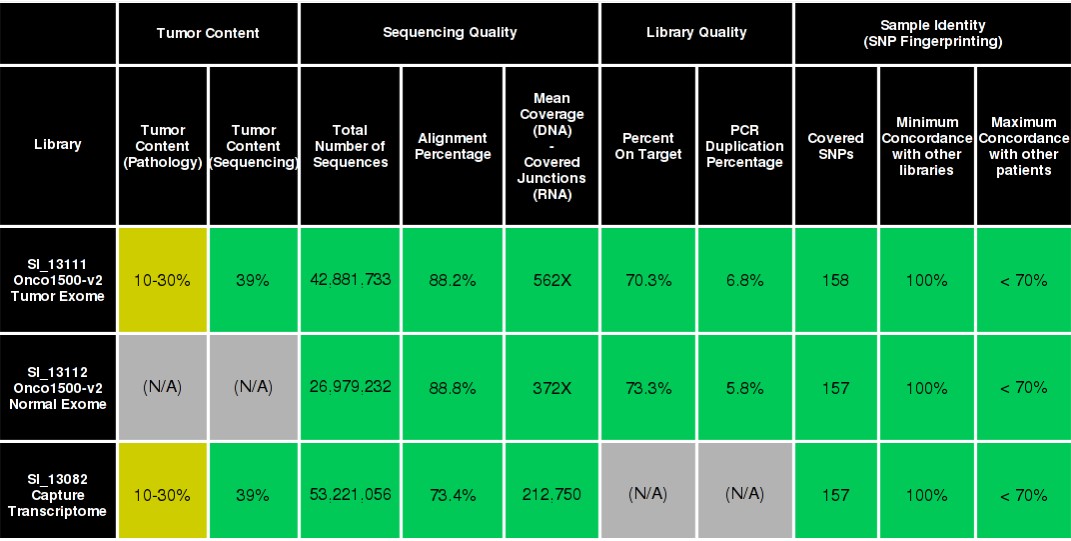


**MO_1582: SEQUENCE ANALYSIS SUMMARY**

***Germline Variations***

| **Gene** | **Location** | **Exon** | **Protein**  **Change** | **dbSNP** | **Variant Reads (Normal)** | **Variant Reads (Tumor)** | **1000**  **Genomes Frequency** | **ExAC**  **Frequency** | **CADD** | **MI-ONCOSEQ**  **Germline Recurrence** |
| --- | --- | --- | --- | --- | --- | --- | --- | --- | --- | --- |
| **PTCH2** | chr1:45295615 | 7 | p.G301S |  | 93/183 (51%) | 139/301 (46%) | 0% | 0% | 25 | 2/1121 |
| **TMC6** | chr17:76113381 | 18 | p.V749A |  | 57/115 (50%) | 76/161 (47%) | 0% | 0% | 18.1 | 1/1121 |
| **WRN** | chr8:31000167 | 27 | p.K1087E | rs374154973 | 128/366 (35%) | 188/518 (36%) | 0% | 0.01% | 0.004 | 2/1121 |
| **TERT** | chr5:1293767 | 2 | p.H412Y | rs34094720 | 80/188 (43%) | 128/272 (47%) | 0.06% | 0.63% | 10.1 | 6/1121 |
| **PALB2** | chr16:23635370 | 8 | p.V932M | rs45624036 | 289/724 (40%) | 440/968 (45%) | 0.10% | 0.60% | 25.8 | 15/1121 |
| **ATM** | chr11:108175463 | 37 | p.D1853V | rs1801673 | 441/441 (100%) | 657/657 (100%) | 0.18% | 0.52% | 19.27 | 16/1121 |


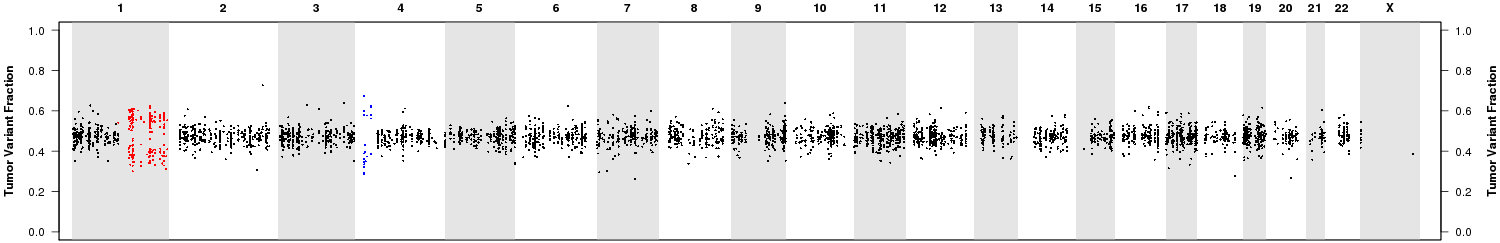


**MO_1582: SEQUENCE ANALYSIS SUMMARY**

***Copy Number Profile & LOH Plot***


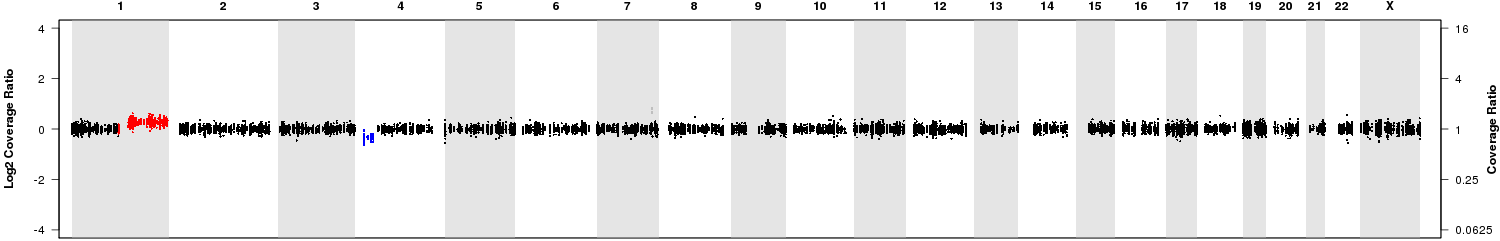


Copy gain: ABL2, AKT3, NTRK1, NOTCH2

Copy loss: FGFR3, GRK4, NKX3-2, SLIT2, WHSC1

***2 Somatic Mutations***

| **Gene** | **Location** | **Exon** | **Effect** | **Protein Change** | **dbSNP** | **Variant Reads (Tumor)** | **Variant Reads (Normal)** | **COSMIC**  **Count** | **CADD** | **FPKM** | **FPKM**  **Percentile** |
| --- | --- | --- | --- | --- | --- | --- | --- | --- | --- | --- | --- |
| **LPHN3** | chr4:62813870 | 14 | Missense | p.R826H | **rs372785017** | 243/1190 (20%) | 3/800 (0%) | **5** | 23.8 | 9.6 | 84.4 |
| **CDH7** | chr18:63491970 | 6 | Missense | p.A295V |  | 194/1085 (18%) | 0/730 (0%) | 0 | 25 | 0 | 46.7 |

LPHN3 (Latrophilin)

**MO_1582: SEQUENCE ANALYSIS SUMMARY**


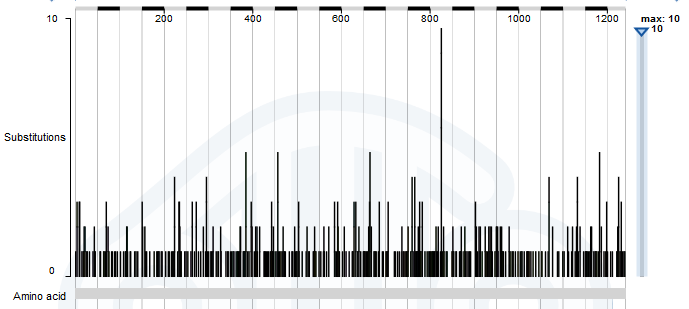


ADGRL3 (Adhesion GPCR L3)

cell adhesion signaling

Proteolytically cleaved into 2 subunits at **aa841-842**: extracellular subunit and a seven- transmembrane subunit.

**liver CRC**

**Prostate glioma melanoma**


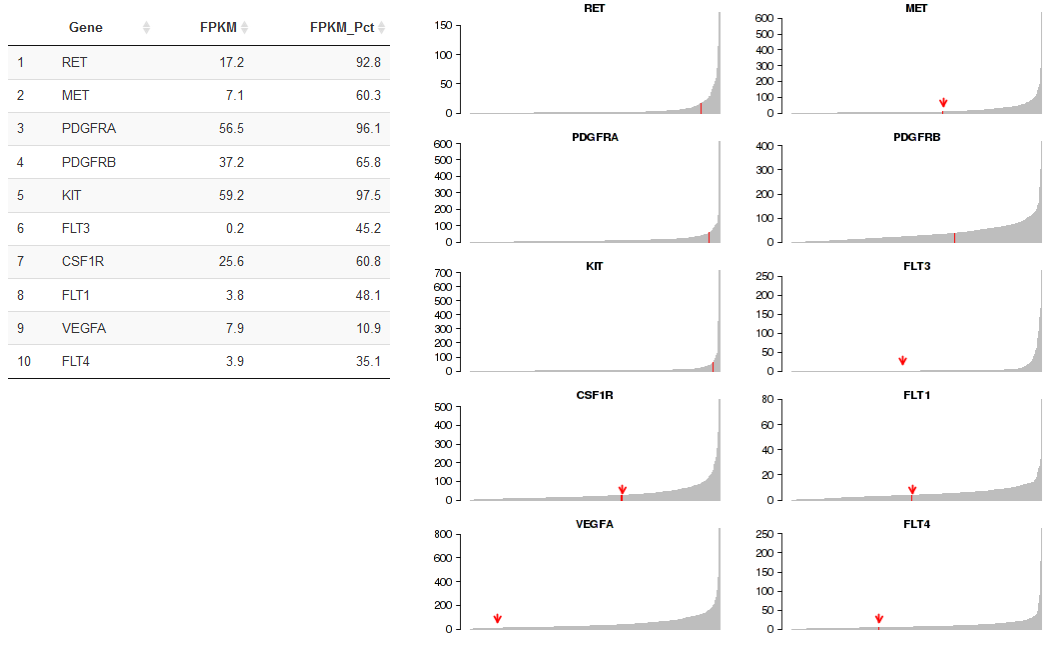


**MO_1582: Disease Biomarkers Expression**

No specific outlier expression

in targetable kinases

**MO_1582: SEQUENCE ANALYSIS SUMMARY**

| **Event/Gene** | **Alteration** | **Comments** |
| --- | --- | --- |
| Extraskeletal Myxoid Chondrosarcoma | Tumor content 39% |  |
| 2 somatic mutations |  |  |
| ***EWSR1-NR4A3*** | **Pathognomonic gene fusion** | associated with sensitivity to Sunitinib |
| ***RET, PDGFRA, KIT*** | Overexpression |  |
| Germline Variants | No Pathogenic germline variants noted |  |
| Pathogens | No pathogens detected |  |
